# Probiotics as technological innovations in psychiatric disorders: patents and research reviews

**DOI:** 10.3389/fnut.2025.1567097

**Published:** 2025-04-24

**Authors:** Alyne Almeida de Lima, Sthefane Silva Santos, Mykaella Andrade de Araújo, Gabriel Vinderola, Cristiane Flora Villarreal, Max Denisson Maurício Viana

**Affiliations:** ^1^Gonçalo Moniz Institute, Oswaldo Cruz Foundation-FIOCRUZ, Salvador, Brazil; ^2^School of Pharmacy, Federal University of Bahia, Salvador, Brazil; ^3^College of Medicine, Federal University of São Francisco Valley, Paulo Afonso, Brazil; ^4^Faculty of Chemical Engineering, Institute of Industrial Lactology, Universidad Nacional del Litoral, Santa Fe, Argentina

**Keywords:** antidepressant, anxiolytic, innovation, patent, probiotics, review

## Abstract

Probiotics have shown promising results in treating anxiety and depression by modulating the gut-brain-microbiota axis using probiotics, which has motivated increasing commercial and academic interest in innovations and the probiotic market. This work explored innovation trends in the use of probiotics in the management of anxiety and depression, through a patent search performed in the Espacenet patent database. To expand the discussion, an additional search was performed on ClinicalTrials.gov and ScienceDirect. Recently probiotic innovations developed were deposited as pharmaceutical products (24.1%), functional foods (20.4%), or both (51.8%). Probiotic strains showed anxiolytic, antidepressant or both effects related to one or more mechanisms including modulation of neurotransmitters (61.1%), neuroendocrine mediators (35.2%) or neuroinflammation and oxidative stress (20.3%). The effects mainly were related to strains of the *Lactobacillus* (48.1%) and *Bifidobacterium* (38.9%) genera. In an additional search, 1,945 scientific publications and 11 clinical trials were found. Despite the efficacy observed in preclinical and clinical studies, transitioning from academic discoveries to patented innovations is not always straightforward. This review provides evidence for therapeutic applications of novel probiotic technologies in treating psychiatric disorders. It supports further studies exploring their benefits and highlights the need for greater investment in innovation in this area.

## 1 Introduction

Globally, psychiatric disorders impact billions of people daily. Among the most prevalent disorders, anxiety and depression grew by more than 25% according to the most recent data (1). Not only due to the pathophysiological confluence, they are similar disorders due to the high rates of disability in the world and have a significant global economic impact on health services of 1 trillion dollars per year (2). Additionally, current treatments have been linked to limiting adverse effects, tolerance, and dependence, which leads to the search for safer therapeutic agents (3). It highlights the urgency to develop economical and accessible strategies to manage depression and anxiety effectively.

Recently, neuroscience research has established the importance of gut microbiota (GM) in behavior and vice versa. This bidirectional communication, the gut-brain-microbiota (GBM) axis, acts mainly through neuroendocrine, neuroimmune, and autonomic nervous system mechanisms (4). In line with these ideas, some authors have demonstrated pattern differences in the composition of the GM among depressive, anxious (markedly more pathogenic), and healthy patients (5, 6). In this sense, psychiatric disorders might be directly associated with perturbations in the microbiota (7, 8). Evidence has suggested that the administration of probiotics, “live microorganisms which, when administered in adequate amounts confer a health benefit on the host,” may improve mental health (9, 10). Clinical and preclinical trials, as well as systematic reviews, have supported this hypothesis. It was demonstrated that probiotic supplementation reduces inflammation and cortisol levels and, consequently stress reactivity, in addition to symptoms of depression and anxiety (10–13). Thus the modulation of the GM using probiotics, represents a therapeutic strategy in the management of depression and anxiety that has aroused academic and commercial interests in recent years.

The global probiotics market is the fastest growing, valued at US$ 87 billion, estimated to be US$ 220 billion by 2030 (14). The increasing interest in probiotics research appears to be related to the abundance of likely advantageous effects in diverse aspects of health and the more lenient rules for registration (15). In this sense, researchers and companies seek to patent their inventions since they can be valuable tools for creating a monopoly and guaranteeing future business in the probiotic market. Recently, many patents have developed new trends for probiotic formulations, mainly aiming to improve the functional properties of probiotics, large-scale production, characterization of therapeutic effects, or even promoting treatment adherence (16, 17). The increasing demonstration of the diversity of sources for obtaining probiotics and developing new formulations and therapeutic applications, especially for mental health conditions, has favored many publications worldwide. In summary, all these studies reinforced the fact that probiotics represent a great source of development of new research aimed at generating innovative products, particular to anxiety and depression conditions with important treatment gaps.

Hence, given the relevance and rise of the proposal, the present work aimed to identify and explore, for the first time, technological trends in treating anxiety and depression using probiotic bacteria through a patent review. Furthermore, the behavioral effects and mechanisms involved in the strains' activity were discussed, emphasizing neural, immunological, and endocrine pathways. To complement the discussion, a search was carried out for scientific articles and clinical trials in the same period. The underlying hypothesis is that the recent increase in patent filings reflects a growing scientific and commercial interest in using probiotic strains to manage anxiety and depression, supported by emerging evidence from neuroscience and microbiology.

## 2 Research reports and patents survey during the period 2003–2023

This is a prospective, descriptive study with a qualitative and quantitative approach. The search was carried out on Espacenet, the European Patent Office (EPO) patent database that includes more than 150 million patent documents from more than 90 countries (worldwide.espacenet.com). The search was carried out through the association of keywords and international patent classification (IPC) related to probiotics, to guarantee the selection of patents in a specific field, namely: “probiotic” AND (“anxiety” OR “depression”) AND (“anxiolytic” OR “antidepressant”) AND (IPC “A61K35/66” [Medicinal preparations containing microorganisms or their materials] OR “A61K35/74” [Bacteria] OR “A61K35/741” [Probiotics] OR ipc = “A61K35/742” OR ipc = “A61K35/744” [Lactic acid bacteria such as *Enterococcus, Lactococcus* and *Streptococcus*] OR “A61K35/745” [*Bifidobacterium*] OR “A61K35/747” [*Lactobacillus* as *L. acidophilus*] OR “A23L33/135” [Bacteria or their derivatives, such as probiotics, in foods]).

**Figure 1:**
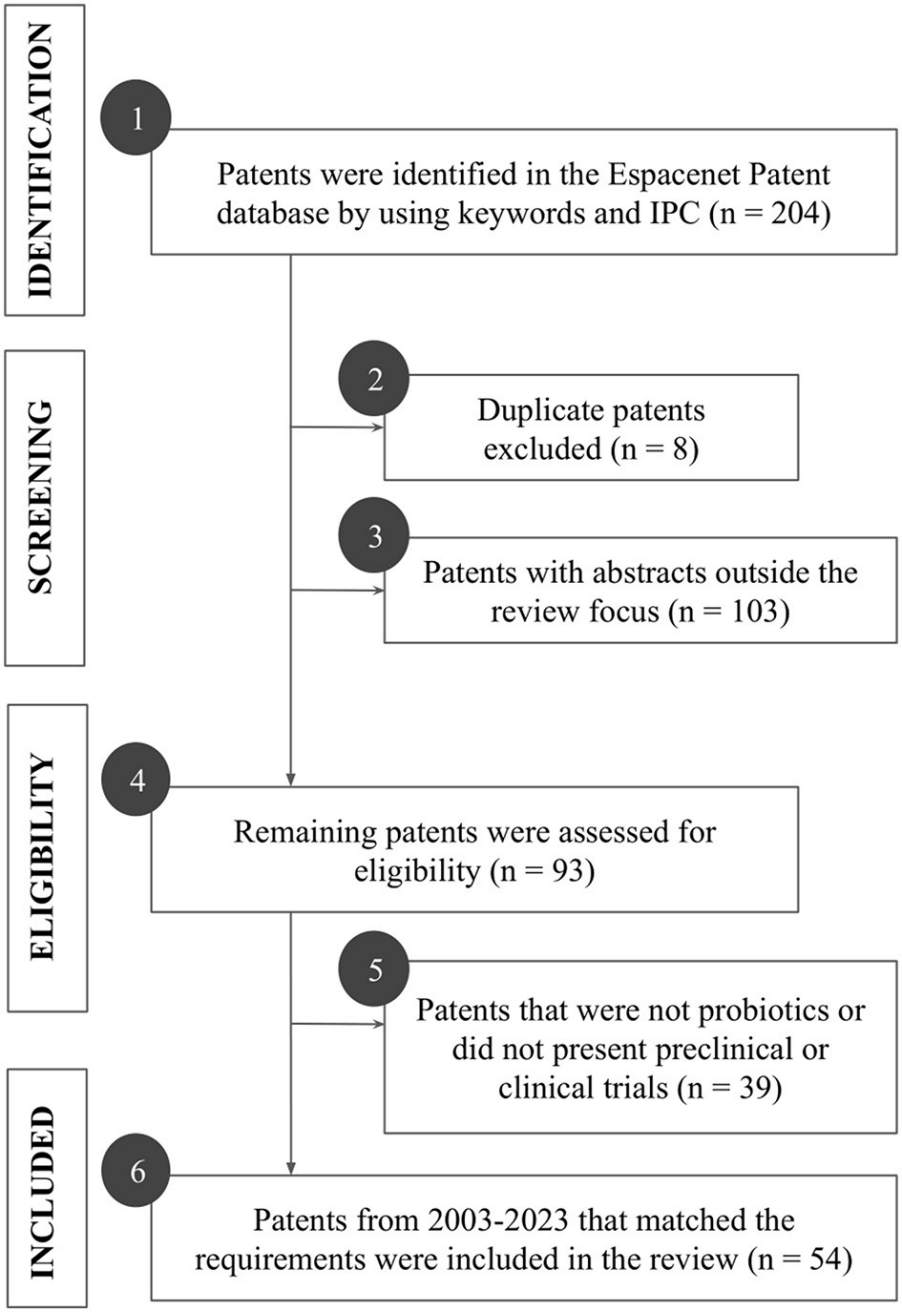
Flowchart of the stages of searching and screening patents for probiotics for anxiety and depression. The patent selection process was represented in four main stages: identification, screening, eligibility, and inclusion. Each action performed in each stage was described in boxes 1–6. IPC: International Patent Classification.

The study only included patents that presented the probiotic's genus, species, or strain; and its effects on psychiatric disorders, specifically on anxiety and depression properly supported by preclinical or clinical trials in appropriate models and tests. Patents that were not available for full reading; patents on probiotic derivatives (prebiotics, postbiotics, paraprobiotics, or synbiotics); patents that did not have the effect of the probiotic strain validated, such as those intended for the treatment of conditions other than the above-mentioned, were excluded. The process of selecting the patents that composed this review is represented in Figure 1. Initially, the search comprised 205 patent filings (Figure 1, box 1). After evaluating duplicated patents (Figure 1, box 2) and reading the abstracts (Figure 1, box 3), 111 patents were removed based on the inclusion and exclusion criteria; then, the remaining 93 patents were fully evaluated (Figure 1, boxes 4,5). Finally, 39 patents were excluded for not reporting on preclinical or clinical trials with probiotics and 54 patents filed from 2003 to 2023 were included in the study and classified by probiotic strain, dose, effects, trials performed, pharmaceutical formulation, year of publication, and country of origin (Figure 1, box 6). Ggplot2 package was used in the RStudio to construct all the graphs.

For additional discussion, the progress of clinical trials aimed at the therapeutic use of probiotics in patients with anxiety and depression, a search was made in Clinical Trial (clinicaltrials.gov). In addition, a screening of scientific publications in the area was carried out in the ScienceDirect database, which has more than 21 million scientific articles published in the most diverse areas, to evaluate whether the scientific publication follows the same patenting standard. The combination of keywords and period used in the patent search were also used in the search for clinical trials and articles in the respective databases. The terms used were “probiotic” AND (“anxiety” OR “depression”) AND (“anxiolytic” OR “antidepressant”), covering publications from 2003 to 2023. However, the IPC was not included as the databases do not support this search strategy. All publications were evaluated together; no screening and eligibility criteria were used.

## 3 Patent filing with emphasis on the management of anxiety and depression

This review initially mapped patents and discussed the data according to the year and country of application. In addition, the innovation potential was presented, and the efficacy profile of probiotic strains was explored with emphasis on the associated mechanisms. Subsequently, an additional discussion was carried out based on the findings in scientific databases and clinical trials.

**Figure 2:**
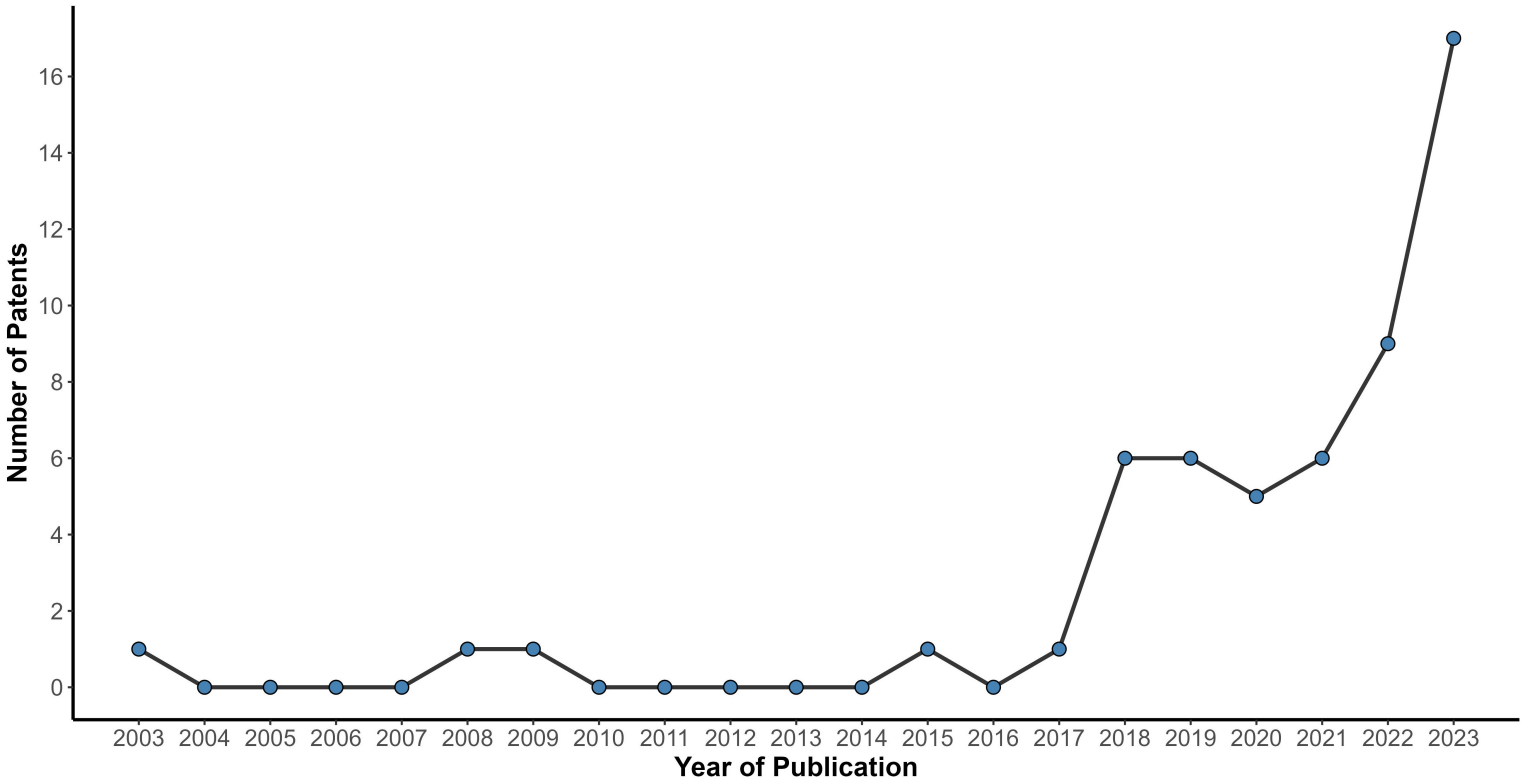
Relationship of the number of patent filed per year (2003–2023). The line chart shows the distribution per year of patent filings from 2003 to 2023. Between 2003 and 2017, the number of filings remained consistently low. From 2022 onward, a sharp rise is observed, culminating in a peak of 17 patents filed in 2023 the highest number of applications.

**Figure 3:**
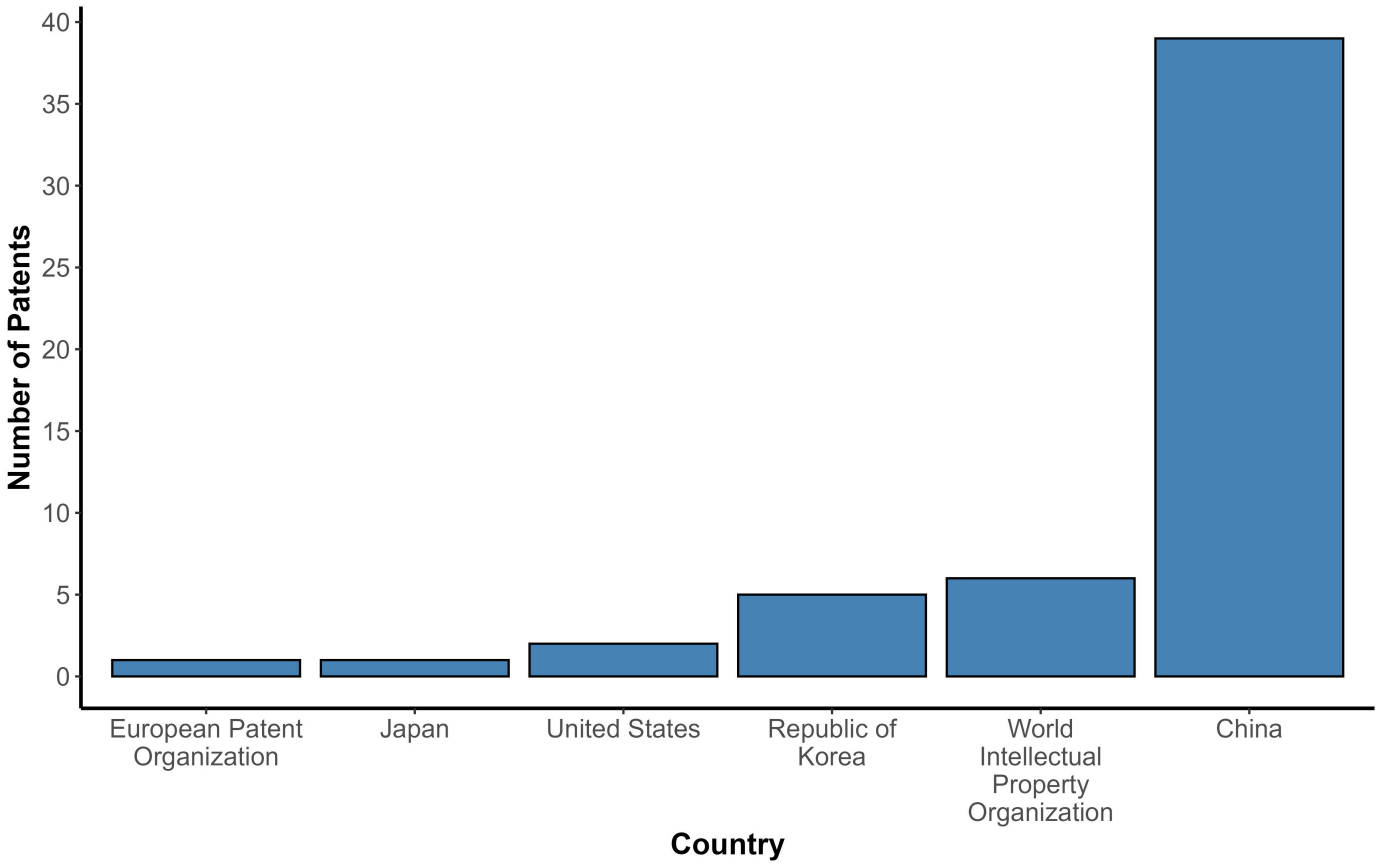
Patent filing by country of origin (applicant). The bar chart displays the number of probiotic-related patents filed by each applicant country. The number of patents is represented on the Y-axis. The countries filing the patents selected for the study are represented on the X-axis. China had the highest number of probiotic patent applications in this study. This distribution highlights geographic disparities.

Figure 2 shows the relationship of the number of patents filed per year (2003–2023). The patent filings remained stable and reduced for 14 years (2003–2017), totaling five patents. However, from 2018 onwards, there was significant growth, reaching around 5 to 6 patents published per year until 2021, followed by nine patent filings in 2022, with a notable peak in 2023 (*n* = 17), the highest number of patents applied in the research period. China led in patent applications (*n* = 39), accounting for more than half of the patents selected in this study (Figure 3). However, this pattern was not observed in other Asian countries, such as Japan and the Republic of Korea, with only 1 and 5 patents filed, respectively. Some patents were filed by the World Intellectual Property Organization (WIPO; *n* = 6), which represents a receiving agency, allowing the inventor to file in a given country(ies) within a 30-month window (18).

The patenting pattern until 2017 remained stable. The WHO report demonstrated that, from 2000 to 2018, 13% of the global population lived with mental disorders, a percentage that also remained stable throughout these years, considering the population increased at the same rate, corroborating our results (1). During this period, research on probiotics was already being conducted in healthy individuals or people with conditions that compromised the microbiota, such as intestinal and respiratory infections, medication use, and psychological stress. The main focus was to restore the balance of the microbiota and the immune system homeostasis. Despite this, the discussion on applying probiotics in treating psychiatric disorders was in its early stages (19), and investments in research and development were still low in this area. Discussions about psychiatric disorders have become more frequent after the COVID-19 pandemic. According to the same report, in 2020, there were 193 million cases of depressive disorders and 298 million cases of anxiety disorders. In 2021, there was 28% and 26% growth, respectively, with 246 million and 374 million cases.

Thus, the applications of probiotics in mental disorders led to an increase in research and development of innovations, observed in 2022 and especially 2023 (18, 20), resulting in new clinical trials and patents in this area (10, 21). The fermented food industry has also been innovating with different probiotic strains, contributing to the increase in patents emphasizing high value-added foods (20, 22).

Despite the interest and investment of other countries in the area, China stands out in this work. China's significant prominence can be explained by the fact that, since the 1990s, the Chinese government has encouraged Chinese inventors to protect their creations through government incentives (23). China's S&T development plans are marked by a long-term goal and an emphasis on innovation and the development of priority areas, including the probiotic products market, with an emphasis on the probiotic-enriched fermented food industry (24).

Furthermore, Asian countries face a significant burden of psychiatric disorders, which are ranked as the eighth leading cause of disease (25). Considering China, a country that presented a significant number of patent filings included in this study, the prevalence of psychiatric disorders is assessed using the China Mental Health Survey created in 2012 at the national level. The compilation of this data until 2015 showed that anxiety disorders were prevalent in 7.6% of the Chinese population. At the time, it was already considered a high prevalence. The authors attribute this increase to improving tracking cases through surveys, interviews with trained professionals, and the rapid economic expansion that implies social changes, generating pressure and stress (26). During the COVID-19 pandemic period, this percentage increased three times, and the prevalence of anxiety was 21.8%. Furthermore, other disorders were also identified, such as depression (26.9%) and stress (48%), demonstrated in a systematic review with meta-analysis of clinical trials evaluated in validated questionnaires (27, 28). These data showed an increase in these disorders, especially during the pandemic, which may reflect the more significant number of patents aimed at such conditions.

After patenting, market entry depends on several factors, such as the presentation of the formulation. Whether in pharmaceutical products or functional foods, the chosen vehicle must maintain the viability of the bacteria in the face of factors intrinsic to the product and the environment, such as pH variations. In addition, it is necessary to comply with specific regulations related to each presentation form. Intended use, target audience, and sensory accessibility, especially in food, are important to offer a good consumer experience and commercial success of the product (29, 30).

The global demand for functional foods is experiencing exponential growth due to technological innovations, new products, and increased health awareness (31). Yogurt, for example, is beneficial in the diet due to its high digestibility and bioavailability of proteins, calcium, potassium, and B vitamins, with a global market estimated at $99,553.38 million in 2019 and projected to reach $141,829.25 million by 2025 (32, 33). Food fortification has effectively prevented micronutrient deficiencies in developed countries, highlighting yogurt for its bioactive peptide content and its adaptability to the addition of prebiotics and probiotics (34).

Functional foods, such as yogurt and other dairy products, contain live microorganisms that benefit the healthy population. The tests must prove the maintenance of physiological functions, configuring a more lenient regulation. While a pharmaceutical product that contains probiotics is called a biotherapeutic product. It is a pharmaceutical product containing live microorganisms applicable to preventing and treating a disease. More rigorous trials are required to prove safety, efficacy, and quality, in addition to a well-defined dosage (35). For clinical trials, enriching dairy products with probiotics presents an advantageous alternative to conventional pharmaceutical forms as it improves patient adherence due to its palatability and serves as a natural carrier that potentially enhances the stability and viability of probiotics in the gastrointestinal tract. This advantage leads to more efficient probiotic delivery and potentially better therapeutic outcomes (34, 36).

Among the patents evaluated, 24.07% (*n* = 13) are applied to pharmaceutical products, and 20.37% (*n* = 11) are applied to functional food. 51.85% (*n* = 28) in both. Only 3.70% (*n* = 2) of patents did not describe the respective applications of the probiotic strain in food or pharmaceutical products. The description of the application of probiotics is more extensive to protect this information. The main foods reported were milk and dairy products, such as yogurt, cheese, and ice cream, as well as tea, soy products, candy, and gum. The leading pharmaceutical formulations involve application in different pharmaceutical forms, such as pills, capsules, and syrup. Regarding the formulation tested in the respective trials described in the patents, the highlights were the probiotic strain in lyophilized powder, 0.9% NaCl vehicle, bacterial suspension in sterile skim milk or phosphate-buffered saline vehicle (PBS), and yogurt.

Most patents protected the applications of the strains in functional food or pharmaceutical products in a broad way and the innovations were the effects presented in the conditions tested; however, the formulation tested for the respective conditions was the main innovation in only two patents. In the clinical trial (CN116676226A) (37), information was provided on the yogurt preparation process, including the probiotic and the experimental design with adult patients with anxiety symptoms. In the preclinical trial (CN111728111A; CN111728111B) (38), the strain was tested in different formulations (solid drink, yogurt, drink, and yogurt block) at different doses. The Preparation of formulations, the incorporation of strains, and experimental design were also provided. All formulations and doses tested showed significantly positive results compared to the control.

As expected, most formulations tested were conveyed in milk and suggested oral administration. Possibly due to the ability of this vehicle to protect bacteria from the acidic environment of the stomach, digestive enzymes, and bile salts, ensuring adequate delivery of a viable quantity of probiotics to the intestine, where they exert their therapeutic action (22). Despite this, maintaining viability is still a challenge, and some alternatives have been proposed in the literature, such as matrix enrichment and microencapsulation of probiotics. Incorporating polyphenol extract from green banana peels into probiotic yogurt increases the viability of *Lactobacillus bulgaricus* and *Streptococcus thermophilus* species without compromising their physical, biochemical, and organoleptic properties. In another study, a millet yogurt formulation with microencapsulated *Lactobacillus casei* resulted in high viability after 28 days of storage at 4°C, although some changes were observed in the sensory analysis (29, 39, 40).

Patent applications also describe probiotics incorporated into ice cream, chocolate, and gum. These applications are also described in the literature, with studies that evaluate the intrinsic parameters of the formulation, such as changes in flavor, color, and resistance to biological barriers, in addition to the therapeutic effects achieved in clinical trials through modulation of the GM. These alternative formulations offer a diversity of consumption options without compromising the viability or effectiveness of probiotics, in addition to allowing probiotics among different target audiences, adults, or children (41, 42).

Regarding the doses tested, the viable number of probiotics described in the patents ranged from 10^6^ to 10^11^ CFU, reflecting considerable dosage variability. Even considering biological barriers, the literature recommends concentrations of 10^6^ to 10^9^ viable probiotic cells to guarantee beneficial effects (43). Yet it is proper to note that no general effective probiotics dose exists. Each probiotic strain is effective at the dose used in the clinical trial where a health benefit was demonstrated, and then that should be the dose delivered by the food or food supplement containing that strain. Recent reviews of clinical trials have pointed out that doses above 10^9^ CFU have been frequently used, with meta-analyses suggesting that doses above 10^10^ CFU were associated with greater therapeutic efficacy in anxiety and depression (44). This reinforces the importance of an adequate dosage for effective therapeutic results.

The complexity of working with live microorganisms, the inadequacy of traditional regulations for synthetic medicines, and different legislation between countries still represent a significant challenge. Therefore, studies are still needed to characterize the effects, doses, and appropriate treatment times, in addition to the appropriate vehicle to maintain several viable cells in developing a biotherapeutic product for the clinical use of probiotics in psychiatric disorders.

### 3.1 Therapeutic potential of probiotics in anxiety and depression and related mechanisms

Depression and anxiety are psychiatric disorders associated with genetic, environmental factors, endocrine and neural dysregulation, inflammation, stress, epigenetic mechanisms and, more recently, evidence reinforces the role of the GM (45, 46). Interestingly, these factors above are directly or indirectly interrelated and have been gaining a better understanding in both psychiatric conditions, especially with the advancement of different technologies for analyzing biological systems, omics sciences, and bioinformatics (47). Studies in experimental models and clinical trials show from the characterization of the microbiota to its modulation (21).

**Figure 4:**
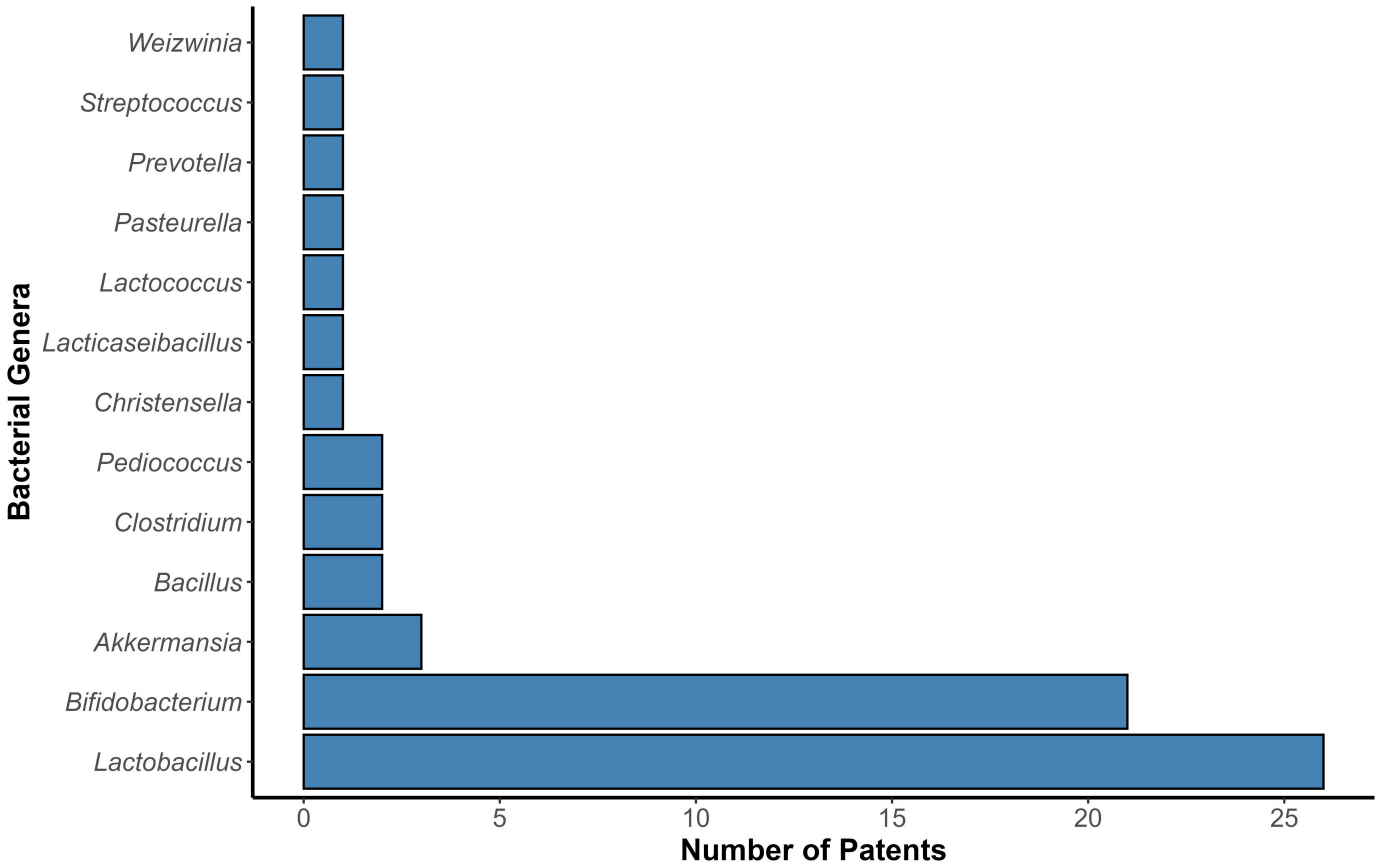
Main probiotic genera described in patents filed for anxiety and depression management. The bars chart presents the number of patents in which each genus was cited. The number of patents is represented on the Y- axis. The probiotic genera described in the patents are represented on the X-axis. *Bifidobacterium* and *Lactobacillus* were the most frequently cited genera described in the patents.

The selected patents explore the action of probiotic strains alone or associated with other microorganisms. Figure 4 shows the main genera of probiotic bacteria used to treat anxiety and depression. Strains of the genus formerly known as *Lactobacillus* (*n* = 26) and *Bifidobacterium* (*n* = 21) genera were the most frequent. Recent research, supported by strong scientific evidence, has highlighted that *Lactobacillus* and *Bifidobacterium* genera are significant modulators of psychiatric diseases by GM pathway (48). Strains of these genera have demonstrated anxiolytic and antidepressant properties, which may be related to by modulating tryptophan levels, hydroxytryptamine (5-HT) synthesis, exerting the anti-inflammatory effects, and the hypothalamic-pituitary adrenal (HPA) axis (49).

Due to these multifaceted interactions, these strains have been increasingly recognized as potential tools in the integrative approach to disorders such as anxiety and depression. The findings described here are consistent with the existing literature that may contribute to the control of behaviors associated with anxiety and/or depression, either by modulation of neurotransmitters, neuroinflammation related to oxidative stress, neuroendocrine modulation, or only based on behavioral tests. Each mechanism was extensively discussed in the following sections. An overview of the probiotic patents studied and their biological effects is provided in Supplementary Table 1.

#### 3.1.1 Neurotransmitters

The pathogenesis of depression and/or anxiety is complex. The imbalance of monoamine neurotransmitters has been the main direction of current research and pharmacological treatments. Studies from the 1960s have already demonstrated that both diseases are closely related to the deficiency of neurotransmitters, mainly 5-hydroxytryptamine (5-HT) or serotonin, in the synaptic cleft (50, 51).

The synthesis of neurotransmitters such as dopamine, norepinephrine, gamma-aminobutyric acid (GABA), and 5-HT can also be modulated by GM and can influence the GBM axis in a complex way, reflecting in higher central functions and the patterns of composition and function of the GM (52). Based on this, systematic review studies with meta-analysis have supported the hypothesis that probiotics can reduce depressive and anxiety symptoms through the modulation of neurotransmitters, suggesting the therapeutic potential of microorganisms (53–56). In the present study, 61.1% (*n* = 33) of the patents evaluated had an influence on neurotransmitter levels mediated by probiotic innovations.

Behavioral models of depression and anxiety in rodents were used to determine the antidepressant and anxiolytic potential of probiotic strains. Among them, open field test, tail suspension test, forced swimming test, sugar preference test, and experiments with buried beads, were employed to test the strains *Lactobacillus plantarum* GM11 (CN115838653A) (57), *Bifidobacterium animalis* subsp. *lactis* HEM20-01 (CN116744806A) (58), *Clostridium* NCIMB 43454 (KR20220119055A) (59), *Weizwinia coagulans* MAT411 (CN116751726A) (60); *Lacticaseibacillus rhamnosus* HN001 (WO2023156945A1) (61); and *Lactobacillus rhamnosus* TF318 (CN108715822A; CN108715822B), CCFM1228 (CN114540245A; CN114540245B), NX-2 (CN117205238A) (62–64). The strains reduced anxious and depressive behaviors, significantly increasing 5-HT levels in brain tissue and blood samples. In the studies, the animals were treated daily for periods ranging from 6 to 40 days, and after the treatment established by the protocol, the samples were analyzed. The dosage of 5-HT in mice treated with the *Lactobacillus paracase*i LC86 strains (CN114774318A; CN114774318B) (65) was carried out through the cerebrospinal fluid, which demonstrated an increase in the concentration levels of 5-hydroxytryptophan (5-HTP), the precursor for the synthesis of 5-HT, and attenuated anxious and depressive behaviors.

The strains *Akkermansia muciniphila* AM06 (CN116474002A), *Lactobacillus pentosus* LPQ1 (CN114410547A; CN114410547B), *Lactobacillus plantarum* R6-3 (CN115948281A) (66–68); *Bifidobacterium animalis* subsp. *lactis GBW8051* (CN116064326A) (69); *Lactobacillus paracasei* nbk-LC16 (CN111560331A; CN111560331B), *Lactococcus lactis* WHH2078 (CN113512514A; CN113512514B), *Bifidobacterium breve* CCFM1025 (CN108949640A; CN108949640B) (70–72); *Bifidobacterium longum* CCFM687 (WO2020037533A1) (73) were also studied separately in rodents using CUMS (Chronic Unpredictable Mild Stress) model, that comprises water and food deprivation, tail pinching, heat stress, cold water swimming, and flash stimulation. The antidepressant potential of the strains was evaluated in open field and elevated plus maze tests, respectively, after chronic treatment, in protocols ranging from 35 to 56 days. Treatment with each strain increased blood serotonin levels and produced therapeutic effects in behavioral tests *Bifidobacterium breve* CCFM1025 (CN108949640A; CN108949640B) (70) was also able to increase the levels of 5-hydroxytryptophan (5-HTP), a direct precursor of 5-HT and serotonin in the prefrontal cortex of rats. Its low bioavailability is associated with a drop in serotonin levels, increasing mood changes in the disease (50).

The strains of *Lactobacillus paracasei* nbk-LC16 (CN111560331A; CN111560331B) (71) also increased norepinephrine and dopamine levels. The antidepressant potential in reducing depression-like behavior was similarly evidenced with the use of the probiotic strain *Christensenella minuta* DSM 3289 (KR20210097716A) (74). The effect was related to an increase in the three neurotransmitters in blood samples, brain, intestine, and fecal content, after 10 weeks of treatment. Levels of 5-HT, norepinephrine, and dopamine in the hypothalamus, thalamus, basal forebrain, and prefrontal cortex regulate memory changes. Furthermore, the hypofunction of norepinephrine and dopamine, especially in the prefrontal cortex, may be related to a lack of energy, which is a recurrent state in depression and anxiety (51).

A probiotic pool containing *Bifidobacterium breve* CCFM1025, *Bifidobacterium longum* subsp. infantile CCFM687, and *Pediococcus lactis* CCFM6432 (CN111743159A; CN111743159B) (75) was also evaluated in depression and anxiety models (sucrose preference, swimming forced, and elevated plus-maze) with daily treatment for 5 weeks. The animals have reduced anxious and depressive behaviors and improved intestinal motility. The patent describes positive therapeutic effects beyond the central nervous system (CNS) area, showing improvement in the GM and highlighting the potential of probiotics to influence various bodily systems. This may align with known biological properties such as modulation of the GBM axis, where changes in the GM impact neurological functions and vice versa, demonstrating the connection between them (52). The measurement in brain tissue indicated an increase in 5-HT concentration. Under similar experimental conditions, *Bifidobacterium bifidum* BXMO (CN113832086A; CN113832086B) and *Lactobacillus gasseri* BDUP (CN116555129A; CN116555129B) (76, 77) were also tested using the elevated plus maze, but to evaluate the neurotransmitter GABA, an inhibitory neurotransmitter often associated to the treatment of anxiety (78). The results indicated that the strains could produce GABA in rodents, as assessed by chromatography and ELISA in samples under the culture condition. GABA is an essential endogenous inhibitory neurotransmitter in the mammalian brain and plays a fundamental role in maintaining the excitatory-inhibitory balance of neural networks (79). The balance between inhibitory GABAergic neurons and excitatory glutamatergic neurons in the brain is crucial for proper brain function. An inadequate supply of GABA can lead to anxiety, fatigue, and distress (78).

The findings on the influence of probiotics on the increase in neurotransmitters and behavioral improvement in animals were not limited to rodent models. This research is still being developed in the zebrafish model. It was observed that *Lactobacillus helveticus* LH05 (CN117379464A) (80) was able to attenuate depressive behavior, in addition to increasing 5-HT concentration levels in brain tissue, in a model of reserpine-induced depression in zebrafish. Corroborating these findings in the zebrafish depression model, the strains *Bacillus subtilis* BS02 (CN117338822A), *Bifidobacterium adolescentis* BAS05 (CN117547558A), *Bifidobacterium animalis* subsp. lactis BL03 (CN117205237A), and *Lactobacillus salivarius* LF01 (CN117398418A) (81–84) showed similar efficacy. The unique characteristics of zebrafish larvae, such as optical transparency, help to elucidate the mechanisms involved in the GBM axis. For example, it is possible to observe labeled bacteria interacting with host cells *in vivo* (85). In zebrafish larvae, anxiety-related behavior is one of the best characterized and is assessed by thigmotaxis, where larvae tend to stay close to the walls of the testing environment, such as a maze or box, rather than exploring more open and exposed areas. This behavior indicates anxiety (86, 87).

Animal trials demonstrate the efficacy and safety of new potential therapies for subsequent clinical studies, which are fundamental for validating and developing new treatments. The transition to clinical trials allows for a more precise assessment of the efficacy and interactions of probiotics with other treatments and health conditions. Additionally, it is essential to consider individual variability and potential adverse effects that may not be evident in animal models (88). The therapeutic effect evidenced by the reduction of depressive behavior and increase in 5-HT levels in brain tissue in rodents by the probiotic pool containing undisclosed strains from the species *Lactobacillus rhamnosus* in a pool with *Lactobacillus acidophilus, Lactobacillus helveticus*, and *Bifidobacterium longum* (CN112999246A) (89) has been supported further clinical studies. Treated patients showed a reduction in scores on the HAM-D (Hamilton Depression Rating Scale) and BDI (Beck Anxiety Inventory) scales. Another important scale, the Antidepressant Side Effects Scale (SERS), was applied before and after treatment with the probiotic pool, resulting in a decrease in adverse effects caused by the use of conventional antidepressants.

Clinical studies with *Lactobacillus plantarum* LP-28 (CN116676226A) (37) were also carried out. 195 patients with anxiety symptoms received probiotic yogurt for 2 weeks. Participants were assessed using the Hamilton Anxiety Index (HAMA) and the BDI psychological scales. After treatment, anxiety levels significantly decreased, although more details on the percentage reduction and statistical significance need to be provided.

Clinical trials, although scarce, have shown that patients with psychiatric disorders have low levels of bacteria that produce beneficial metabolites and low anti-inflammatory potential (90, 91), favoring neuroinflammation. The immune response triggered may influence other components relevant to neuropsychiatric conditions, such as neurotransmitter metabolism, since interleukin-1 beta (IL-1β) and tumor necrosis factor-alpha (TNF-α) can inhibit the release of 5-HT and dopamine, while IL-6 can increase the release of glutamate (11). Therefore, inhibiting the production and release of such mediators, favoring neurotransmission, may be an important strategy in the management of psychiatric disorders.

#### 3.1.2 Neuroinflammation and oxidative stress

Several studies and reviews have demonstrated a significant link between chronic inflammation, anxiety, and depression (92). The differential expression of inflammatory markers, such as pro- and anti-inflammatory cytokines, in the peripheral context and/or in the CNS, reinforces this association, as reported in animal models and clinical data (93). Among the main molecules reported in both conditions are IL-6, TNF-α, and IL-1β. Furthermore, patients treated with antidepressants have lower levels of pro-inflammatory cytokines (90). In addition to inflammation, oxidative stress is another critical mechanism. The accumulation of reactive oxygen species (ROS) increases blood-brain barrier permeability, contributing to brain pathophysiology (94). Oxidative stress is implicated in depression, marked by elevated biomarkers (95), and gene expression changes that increase oxidative stress in anxiety models (96). Targeting oxidative stress pathways is suggested for developing new psychiatric treatments. Probiotic strains have demonstrated therapeutic effects in regulating inflammation and/or oxidative stress in modulating anxiety and depression (97, 98). These properties were observed in 20.37% (*n* = 11) of the evaluated patents.

*Bifidobacterium infantis* 35624 (WO2004098622A2; WO2004098622A3) and *Bifidobacterium adolescentis* IM38 (KR101862051B1) (99, 100) strains have demonstrated anti-inflammatory effects by reducing proinflammatory markers in clinical and pre-clinical trials, respectively. The strains of the first species showed an antidepressant effect in humans, as evaluated by HAMD and BDI scores when administered for three weeks. Reduced Soluble Interleukin 6 Receptor (sIL-6R) and IL-8 levels were also observed in blood samples. SIL-6R is required for IL-6 trans-signaling, one of three distinct modes of signaling (101). The later strain, *B. adolescentis* IM38, was tested for anxiety and depression in C57BL/6 mice. Using the elevated plus maze, treated mice exhibited reduced anxiety-like behavior and lower IL-6 and TNF-α systemic levels in blood samples.

Some probiotics have exhibited molecular effects, not only addressing neuroinflammation and/or oxidative stress but also modulating neurotransmission, as discussed in the previous section, and by influencing the HPA axis, which will be discussed in the following section. In a preclinical model of CUMS, strains of *Lactobacillus pentosus* LPQ1 (CN114410547A; CN114410547B) (66) were administered at a dose of 1 × 10^9^ CFU/mL for five weeks. *Pediococcus acidilactici* CCFM6432 (WO2020238870A1) (102) was also tested in the context of animal models of CUMS which were treated with 5 × 10^9^ CFU/mL for 5 weeks. In addition to analyzing depressive effects as in the previous patent, here, anxiety assessments were also conducted, such as the elevated plus maze test. Notably, animals treated with the probiotic exhibited significant effects in both tests, with effects superior to those of fluoxetine. In the context of inflammation, decreased levels of pro-inflammatory cytokines IL-6, IL-1β, and/or TNF-α in serum were also observed.

Behavioral analysis of probiotic-treated animals using sugar water preference, forced swimming, tail suspension, and open field tests showed an attenuation of depressive-like or anxiogenic behaviors. Interestingly, molecular analysis indicated decreased levels of proinflammatory cytokines, similar to the effects on neuroinflammation that have been previously described (51). Through evaluation in these preclinical models, certain strains demonstrated unique antidepressant and/or anxiolytic effects by modulating all three main molecular targets of this review (neurotransmitters, neuroinflammation/oxidative stress, and neuroendocrine modulation), namely: *Akkermansia muciniphila* AM06 (CN116474002A), *Lacticaseibacillus rhamnosus* HN001, and/or *Bifidobacterium animali*s subsp. *lactis* HN019 (WO2023156945A1) and *Lactobacillus plantarum* GM11 (CN115838653A), *Lactobacillus plantarum* R6-3 (CN115948281A), *Lactobacillus rhamnosus* KY16 (CN117143760A), *Lactobacillus rhamnosus* TF318 (CN108715822A; CN108715822B), and *Bifidobacterium animalis* subsp. *lactis* HEM20-01 (CN116744806A) (57, 58, 61, 62, 67, 68, 103).

Administration of the first four strains significantly suppressed the pro-inflammatory markers TNF-α, IL-1β, and IL-18 in brain tissue and blood and IL-6 exclusively in blood samples. For *L. plantarum* R6-3 (CN115948281A) and *L. rhamnosus* KY16 (CN117143760A) (68, 103) strains, there was an improvement in the state of immunological and oxidative stress by the reduction of IL-1β and IL-6, and upregulation of IL-10, an anti-inflammatory cytokine, and specific increase of antioxidant markers such as glutathione peroxidase (GPX), superoxide dismutase (SOD), total antioxidant capacity (T-AOC), nuclear factor erythroid 2–related factor 2 (Nrf-2), and decrease of malonaldehyde (MDA) after treatment with the first strain.

In addition, *L. rhamnosus* KY16 treatment was able to upregulate genes indicative of M2 polarization of glial cells. The reduction of proinflammatory cytokines was also evidenced by *L. rhamnosus* TF318 (CN108715822A; CN108715822B) (62) strains, as well as the reduction of markers of oxidative stress as nitric oxide (NO) and lipid peroxidation as MDA in the blood. Post-treatment analysis still revealed significantly lower levels of pro-inflammatory cytokines (IL-1β, IL-6, TNF-α, and Interferon-γ) in the ileum samples and the central microenvironment (hippocampus) after treatment with *Bifidobacterium* strain.

The imbalance between the production of ROS and antioxidant enzymes is termed oxidative stress. Optimal amounts of these enzymes play an important role in restoring homeostasis. SOD, for example, catalyzes the conversion of the superoxide anion into hydrogen peroxide, which is further converted into water and oxygen by either catalase or GPX. Interestingly, transgenic mice overexpressing SOD1 exhibited greater resistance to corticosterone-induced depressive-like behaviors compared to wild-type animals (104). Additionally, these animals had fewer oxidative stress markers in the hippocampal CA3 region. This indicates that SOD1 overexpression protects against depressive behaviors by reducing cellular ROS.

Nrf-2 regulates the activation and expression of the antioxidant response element by positively regulating this activity, which enables the expression of antioxidant genes, including NADPH-dependent antioxidant enzymes (105). Deficient Nfr2 plays a significant role in oxidative stress in animals (106), and small interfering RNA (siRNA) against Nrf2 in rats promoted greater anxiety-like behavior concerning the control group (107). Indeed, patients with major depression present lower levels of this mediator (108). The Modulation of Nfr2 may be an interesting strategy for the therapeutic approach to both depression and anxiety disorders.

In the context of lipid peroxidation, MDA is formed through a reaction of free radicals with unsaturated fatty acids such as Omega-6 and−3, which are components of neuronal membranes. It has been demonstrated that patients with major depressive disorder have significantly higher plasma levels of MDA compared to healthy individuals (109). Treatment with selective serotonin reuptake inhibitors (SSRIs) such as citalopram and fluoxetine significantly reduced serum MDA levels (110).

For example, the polarization of M2 state via IL-4 or IL-13 initiates the activation of STATt3, a critical transcription factor that generates a specific gene expression pattern. These M2 macrophages facilitate the resolution of inflammation through anti-inflammatory factors, such as IL-10 and TGF-beta, Arg1 and others, which deactivate pro-inflammatory cell phenotype, corroborating the homeostasis context (111).

Consequently, modulating inflammation, oxidative stress, and lipid peroxidation, along with the aforementioned, are strategies that may contribute to the management of depression and anxiety. These mechanisms have complex and interdependent interactions that significantly affect neuroendocrine regulation, particularly the HPA axis, as described below.

#### 3.1.3 Neuroendocrine modulation

The HPA axis is a hormonal signaling cascade involved in adaptive and emotional responses. Chronic activation of the axis due to psychological stressors results in altered expression of factors, such as brain-derived neurotrophic factor (BDNF), and altered levels of hormones, such as cortisol. Among the consequences of this hyperactivation is the development of psychiatric disorders, possibly associated with dysregulated negative feedback (28, 112). Therefore, the neuro-immunomodulatory activity of probiotic strains in modulating hormonal levels (cortisol) and factors associated with the CNS may be an important strategy for anxiety and depression management. Among the patents evaluated, 35.18% (*n* = 19) were applied in this context.

The effects of some strains have been associated with an increased expression of hippocampal BDNF on anxiety and depression in different protocols in mice treated with *Clostridium* NCIMB 43454 (KR20220119055A) (59) for six days. *Bifidobacterium longum* ATCC BAA-999 (EP3072398A1; EP3072398B1) (113) significantly reduced only anxiety-like behavior for ten days. While *Bifidobacterium longum* subsp. *infantis* CCFM687 (WO2020037533A1) (73) only reduced depression-like behavior when administered for four weeks. BDNF is an important neurotrophin expressed in several brain regions. It is involved in signaling pathways promoting neurogenesis and neuroplasticity, especially when expressed in the hippocampus. However, in individuals with psychiatric disorders, a reduction in BDNF levels is observed, possibly due to changes in its genetic expression and the occurrence of polymorphisms in coding regions (114, 115). The GM plays an important role in increasing BDNF levels, possibly by stimulating the vagus nerve. In vagotomized mice and treated with the *Bifidobacterium longum* NCC3001 strain, there was no reduction in anxiety-like behavior (116, 117). A systematic review showed that germ-free mice had reduced hippocampus neurogenesis and BDNF mRNA expression. In contrast, analysis of the taxa involved in control mice demonstrated a more positive correlation of neurogenesis and expression of *Lactobacillus* and *Bifidobacterium* (118). Surprisingly, the majority of probiotic patents included in this review belong to these taxa and this effect has been demonstrated in the literature as one of the mechanisms responsible for reducing anxiety- and depression-like behaviors in rodents (119, 120).

Other patents presented effects associated with the increase of BDNF, such as modulation of the HPA axis by altering the levels of important corticotrophins, as adrenocorticotropic hormone (ACTH) and corticotropin-releasing hormone (CRH), and corticosterone (or cortisol), which were mainly associated with reduced depression-like behavior. Treatment with a single dose of *Lactobacillus plantarum* DP18 (CN110066753A; CN110066753B) (121) in rats was able to reduce depression-like behavior, which was associated with an increase in BDNF expression in the hippocampus and a reduction of ACTH in serum. *Lactobacillus rhamnosus* CCFM1228 10^9^ CFU (CN114540245A; CN114540245B) (63) reduced CRH and corticosterone, and increased BDNF expression in mice after eight weeks of treatment. *Lactobacillus plantarum* R6-3 10^8^ CFU (CN115948281A) (68), and different strains of *Bifidobacterium*: *Bifidobacterium breve* CCFM1025 5x10^9^ CFU (CN108949640A; CN108949640B), *Bifidobacterium animalis* subsp. *lactis* GBW8051 10^10^ CFU (CN116064326A), and *Bifidobacterium animalis* subsp. *lactis* HEM20-01 5x10^7^ CFU (CN116744806A) (58, 69, 70), reduced serum corticosterone levels and also increased BDNF expression in the brain tissue of mice. Through this same mechanism, *Lactococcus lactis* WHH2078 (CN113512514A; CN113512514B) and *Lactobacillus plantarum* GM11 (CN115838653A) (57, 72), both 10^9^ CFU, reduced anxiety- and depression-like behaviors in mice.

Increased cortisol levels in patients with psychiatric disorders are associated with the severity and type of disorder and are more common among depressive patients. In these cases, hypercortisolemia indicates the recurrence of the condition with an unfavorable prognosis. This hormone is mainly released in acute response to stress (122, 123), which makes it an important target in the development of new strategies for the management of mental disorders. CRH and ACTH are important hormones secreted into the bloodstream when a stressful stimulus activates the HPA axis and precede the release of cortisol (124). CRH is produced and secreted by neurons in the hypothalamic paraventricular nucleus (PVN), a site involved in gastrointestinal responses triggered by stress. The PVN is the main center for relaying signals from the vagus nerve through afferent pathways (125, 126). Mice treated with the *Lactobacillus* strain demonstrated a reduction in anxiety- and depression-like behaviors and corticosterone levels. However, vagotomized mice treated with the same strain did not demonstrate a reduction in these parameters (127). Therefore, just as the increase in BDNF expression by probiotic bacteria is mediated by stimulation of the vagus nerve, evidence indicates that this mechanism is also related to the increase in cortisol.

For other patents, neuroendocrine activity occurred only through modulation of the HPA axis, as an additional effect to the effects already described in the previous sections. *Akkermansia muciniphila* AM06 (CN116474002A) (67) was shown to modulate the HPA axis by reducing CRH levels and anxiety- and depression-like behaviors in mice treated with 10^10^ CFU for 56 days. Also, rodents treated with *Pediococcus acidilactici* CCFM6432 5x10^9^ CFU for five weeks (WO2020238870A1), *Lacticaseibacillus rhamnosus* HN001, and *Bifidobacterium animalis* subsp. *lactis* HN019 (WO2023156945A1) (61, 102), alone or in combination, for 40 days, demonstrated a significant reduction in serum corticosterone levels. Through this exact mechanism, *Lactobacillus rhamnosus* KY16 (CN117143760A) and *Lactobacillus rhamnosus* TF318 2x10^10^ CFU (CN108715822A; CN108715822B) (62, 103) only reduced depression-like behavior in mice treated for 5 weeks and 21 days, respectively. A probiotic pool composed of *Bifidobacterium breve* CCFM1025, *Bifidobacterium longum* subsp. *infantiles* CCFM687, and *Pediococcus lactis* CCFM6432 (CN111743159A; CN111743159B) (75), was also able to reduce blood ACTH, in addition to blood corticosterone levels, while reducing depressive-like and anxiety-like behaviors in mice treated for 5 weeks with 10^9^ CFU. While *Lactobacillus paracasei* nbk-LC16 10^8^ CFU (CN111560331A; CN111560331B) (71) reduced CRH and corticosterone, concomitantly with the reduction of depression-like behavior in rats after 4 weeks.

In this review, reducing cortisol (or corticosterone) and increasing BDNF were identified as targets in probiotic therapy for anxiety and depression. However, in the literature, there are still discrepancies about this. A systematic review and meta-analysis of clinical trials with patients with psychiatric disorders reported a significant increase in BDNF levels in individuals treated with probiotic strains, who also showed symptomatic reduction. However, no significant results were observed in the reduction of cortisol (128). While the strain investigated by Lee et al. (129) did not promote increase of BDNF expression nor reduced cortisol levels. But it reduced anxiety and depression scores in symptomatic patients after probiotic intervention. The main differences are associated with the strain, dose, and collection time, which influence cortisol levels.

These effects have not been described in isolation. Pharmacological approaches in the chronic treatment of psychiatric disorders mainly aim to increase 5-HT. However, evidence suggests that there is also an increase in neurogenesis mediated by BDNF expression. Depletion of certain 5-HT receptors reduces BDNF expression and antidepressant behavior. It is also suggested that the latency of effects is due to the late onset of neurogenesis (114). Furthermore, chronic activation of the cortisol release cascade (by HPA axis) results in pathological responses, such as increased intestinal permeability to pro-inflammatory mediators (123). Therefore, some probiotic strains were able to modulate these mechanisms simultaneously, as they are interconnected. This opens up gaps for future studies on the choice and combination of appropriate bacteria and probiotic doses.

#### 3.1.4 Just behavior

The anxiolytic and antidepressant potential was also demonstrated only through behavioral models or questionnaires. It represented 29.63% (*n* = 16) of patents. Preclinical models are based on models of approach and exploration behavior and avoidance, which are animals' natural behaviors. There are models called ethological, based on the exploration/avoidance of unknown environments, as animals naturally avoid new, illuminated, open, and elevated places. And there are conditioned models, in which the response is presented when conditions of fear or changes in eating patterns are introduced and require the training of the animals (130–132).

In parallel, human assessments count on standardized questionnaires to evaluate the efficacy of therapeutic interventions in anxious and depressive-like behaviors such as Hamilton Depression Rating Scale (HAM-D), State-Trait Anxiety Initiative (STAI), Hamilton Anxiety Index (HAMA), Leiden Index of Depression Sensitivity-Revised (LEIDS-R), and 62-question questionnaire on depressive symptoms. These tools are critical in quantifying the severity or specific features of anxiety and depression, corroborating findings observed in animal models.

Preclinical studies were conducted to evaluate the effects on concomitant anxiety- and depression-like behaviors. *Akkermansia muciniphila* JWA48 (CN113322202A; CN113322202B) (133) at 1x10^9^ CFU increased the total movement distance and time in the central area in the open field test, parameters related to anxiolytic effects. In the tail suspension test, C57BL/6 mice treated with the same strains showed shorter immobility time, suggesting an antidepressant-like effect. *Lactobacillus plantarum* strains LP12151, LP12418, and LP12407 (KR20200101950A) (134) were tested in *Swiss* mice subjected to the chronic stress model. The animals were placed in transparent tubes under bright light for 180 minutes daily, five days a week for 3 weeks. After treatment for 33 days at 1x10^9^ CFU, an increase in entries into the open arms (only for LP12407 and LP12418) in the elevated plus maze test and a reduction in immobility time in the forced swimming test were observed, suggesting anxiolytic and antidepressant potential.

*Bifidobacterium* AH171 (US11225641B2; US2020325548A1) (135) also demonstrated antidepressant effects, reducing depressive-like behavior in the tail suspension test. After 3 weeks of treatment, mice also showed a reduction in anxiety-like behavior in the fear conditioning test, which promoted learning and contextual fear memory, and in the marble burying test, they buried fewer marbles. The animals treated with the probiotic showed similar results to those treated with escitalopram, a SSRI drug widely used in the clinic. In addition to presenting effects in isolation, the combination of strains of *Lactobacillus* and *Bifidobacterium* resulted in a new product called K-KLJ (CN111728111A; CN111728111B) (38). Mice treated with a probiotic combination in yogurt at 10^8^, for 30 days, remained longer in the light place in the light-dark box and reduced immobility time in the tail suspension test, which points to effects on both psychiatric conditions.

A single patent was associated only with anxiety. The combination of *Lactobacillus rhamnosus* and *Lactobacillus fermentum* (CN114947135A; CN114947135B) (136) was able to reduce anxiety-like behavior in mice at 1 × 10^8^ CFU. The majority of these patents evaluated depressive-like behaviors and associated anhedonia. The main preclinical tests were the tail suspension test and forced swimming, which are based on “behavioral despair”. To assess anhedonic behavior: sucrose preference, spray, platform, marble-burying, water maze, and social interaction tests, which are based on the reduction of pleasure, self-care, and social interaction in rats and mice. The clinical trials on depressive patients treated with the probiotic strains were conducted by these questionnaires: LEIDS-R, STAI, HAMA, and 62-question questionnaire, which will be next described.

Although the probiotic strain is not disclosed, CN112914107A; CN112914107B (137) is an Ls17 probiotic that has shown promising antidepressant effects. The strain was tested in germ-free mice, which subsequently received fecal microbiota transplants from depressed patients. BALB/c were treated with a probiotic dose of 1 × 10^9^ CFU. Also, *Akkermansia muciniphila* (WO2021008149A1) (138) was tested after fecal microbiota transplantation from depressed patients into C57BL/6 mice. In addition to the transplant, the animals were subjected to a chronic restraint stress model in tubes for 3 hours every day for 30 days to establish depressive-like behavior. After treatment, there was a reduction in depression-like behavior. *Pasteurella enterica* DSM 21032 (CN117530964A) and *Lactobacillus animalis* JCM 5670 (CN116327813A) (139, 140) were tested on the CUMS model em C57BL/6J mice and SD rats, respectively, both at 10^9^ CFU, and resulted in a reduction in the depression parameters assessed. At the same dose, *Lactobacillus plantarum* LP12151 (KR20210102916A) (141) was tested in animals after 30 days of treatment in the chronic stress model previously described. After treatment, Swiss mice showed a reduction in immobility time, characterizing a reduction in depression-like behavior. The combination of *Lactobacillus plantarum* Lp3a, *Lactobacillus paracasei* LPC45, and *Bifidobacterium breve* BB033 (CN114081184A; CN114081184B) (142) at a dose of 2 x 10^11^ CFU was tested in mice, and showed an antidepressant effect. Another combination was tested at a dose of 10^10^ CFU in C57B1/6 mice. *Lactobacillus salivarius, Lactobacillus camelliae*, and *Bifidobacterium ruminantium* (WO2023079036A1) (143) had its antidepressant effect associated with oxytocin-like activity.

*Bifidobacterium bifidum* W23 (US2018271919A1) (144) has been tested in rats and healthy humans. The animals were treated for 8 weeks and showed reduced depression-like behavior. Patients were treated for 4 weeks and evaluated by LEIDS-R. The questionnaire reflects cognitive reactivity in response to bad mood, that is, the tendency to think negatively when in a sad mood, a marker of vulnerability to depression. At the end of treatment, scores decreased significantly in the probiotic group in the aggressiveness and ruminative thinking categories.

Among the technologies evaluated in clinical trials in depressive patients, two were combinations of probiotic strains. CN110279119A (145) is a pool probiotic (called Nagqu 4580) of *Streptococcus thermophilus* S709, *Lactobacillus paracasei* L578, and *Lactobacillus helveticus* L551. Participants were treated for 2 weeks. After treatments, the HAMA questionnaire score significantly decreased in patients treated with the probiotic pool at a dose of 1 × 10^9^ CFU, which suggests an antidepressant effect. While CN117297098A (146) is an association of *Bacillus Coagulans, Bifidobacterium breve, Bifidobacterium Infantis, Bifidobacterium longum, Lactobacillus casei, Lactobacillus helveticus, Lactobacillus paracasei, Lactobacillus plantarum*, and *Lactobacillus rhamnosus*. The 62-question questionnaire (A1, A2, and A3) was applied at different time scales during the treatment (10^10^ CFU). Initially, the patients received probiotic supplementation twice a day, and assessments took place before supplementation (A0); in the 3rd week (A1); administration became once a day in the 4th week (A2); and once a day until the 6th week (A3). The depression score on the questionnaire was significantly reduced in A3 compared to the untreated depressive group.

Singly, *Lactobacillus herbeticus* NITE BP-01671 (JP2021045054A) (147) at a dose of 10^10^ CFU was developed for anxiety and depression. The evaluation was taken by the STAI, which is used to measure depressed mood and anxiety. After 8 weeks of treatment, the score significantly decreased in the group treated with the probiotic strain.

Behavioral analysis is crucial for understanding the therapeutic potential of probiotic strains in depression and anxiety. However, it is insufficient to determine the physiological effects driven by molecular alterations, which are necessary for a more comprehensive understanding of probiotic strain's role in modulating the diseases. Moreover, this intersection reinforces the importance of integrating behavioral and biomolecular analyses to elucidate the underlying mechanisms contributing to the observed benefits of probiotics in both psychiatric conditions.

## 4 Relationship among patent deposits, scientific publications and clinical trials

### 4.1 Scientific publication

**Figure 5:**
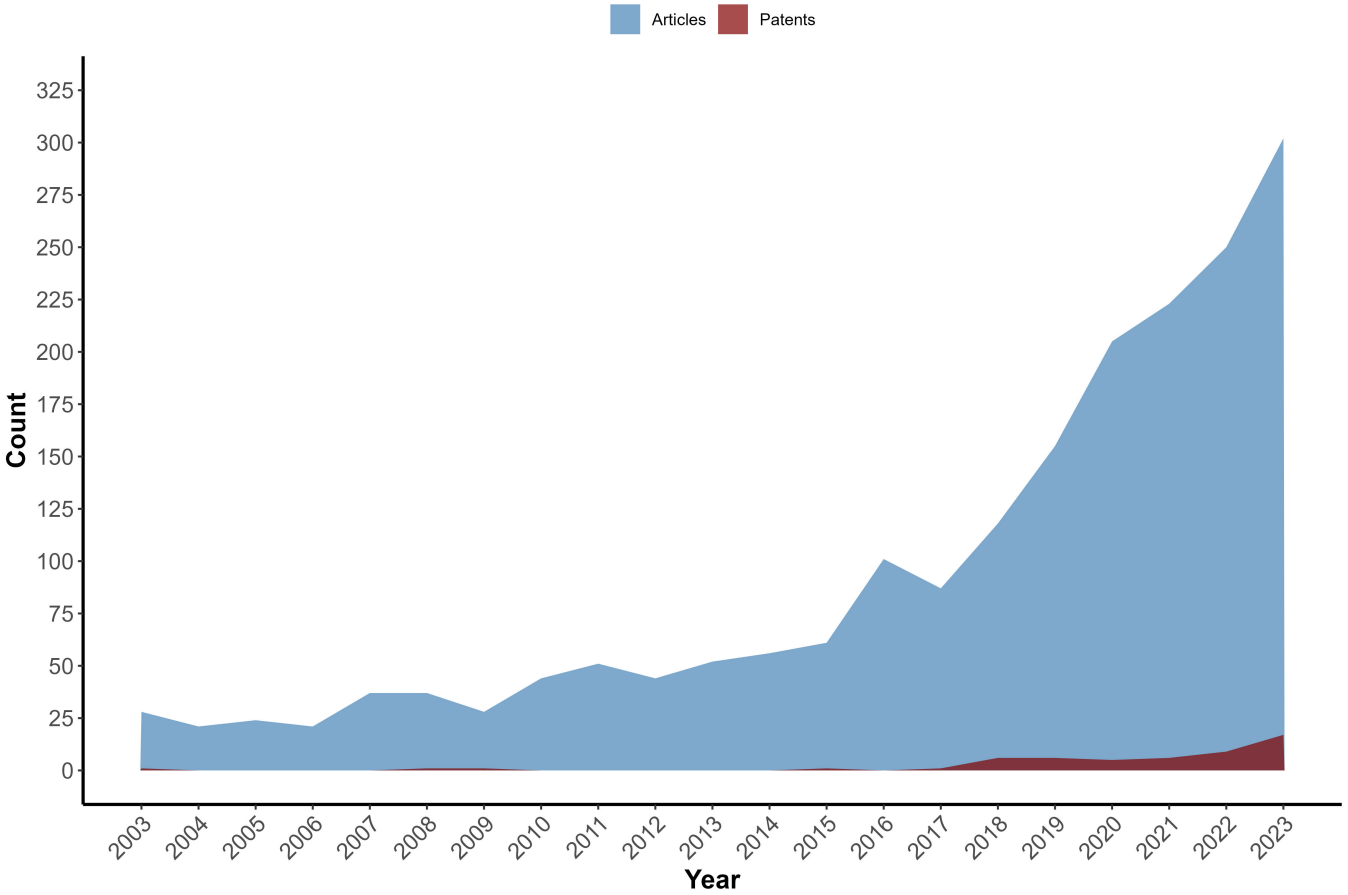
Evolution of the number of scientific article publications and patent filings (2003–2023). The area chart displays the annual count of scientific articles (blue areas) and patent filings (dark red) over the years, with the X-axis representing time (2003-2023) and the Y-axis representing the total number of records. Throughout the period, the number of scientific articles is consistently higher than that of patent filings.

The total number of patents filed between 2003 and 2023 was lower than the number of scientific articles published in the same period. The complementary search in the ScienceDirect database returned 1945 studies (Figure 5). In some of the periods researched, patents were not registered. Among the studies, 621 (31.93%) review articles and 529 research articles (27.20%) were found, in addition to book chapters, case reports, and others (40.87%). The same profile was observed in another review of probiotic patents for other conditions (17), with an increased number of articles published compared to patent filings in the same period.

It is also important to highlight that, in certain countries, legislation prohibits patenting microorganisms, which reinforces the relevance of prioritizing the publication of scientific articles over patent registrations as a strategy for disseminating knowledge. Even among highly productive scientists in science and technology, the number of articles published is greater than the number of patents granted (148). Both authors attribute such differences to the purpose of each publication and its impact since patenting requires greater rigor. In contrast, the number of publications and quantified citations are still performance indicators. Furthermore, not all publications present promising results for applying for a patent. This suggests that, although research and development in specific areas such as probiotics are growing, the transition from academic discoveries to patented innovations is not always straightforward.

The need for rigorous validation and robust evidence for patents can limit the ability to transform scientific publications into marketable products quickly. This situation reinforces the importance of fostering collaborations among industries, regulatory agencies, and researchers to conduct preclinical and clinical studies, facilitating technology transfer and protecting promising innovations. Thus, the disparity between the number of articles and patents highlights the complexity of the innovation process and the challenge of translating discoveries into practical applications in the market (149).

### 4.2 Clinical trials

Eleven studies were found in Clinical Trials. The distribution in different countries is relatively balanced, demonstrating global participation in clinical research. Brazil, Poland, the United Kingdom, Switzerland, the United States, and France each have a registered clinical trial. While Canada and Taiwan stand out with two clinical trials each. Only one of the studies did not disclose its location. Five completed trials were found regarding the phases of the trials, three with unknown progress status, two recruiting, and one not yet recruiting. Surprisingly, China, the most significant patent applicant in the present study (n = 39), does not appear among the countries that advanced from preclinical to clinical studies. Indeed, some authors have pointed out some difficulties or quality problems of clinical trials in China. The problems reported include ethical review, registration, implementation, and poor standardized supervision systems (150).

All trials were conducted with patients with major depressive disorder who were already using conventional treatment, mainly SSRIs. Possibly because studies suggest a greater change in microbiota diversity in patients with depression (151). They presented a similar experimental design, with 4, 8, and 16 weeks of oral treatment, administered once, twice, or four times daily. The literature indicates that oral treatment with probiotics to alleviate symptoms of depression should last more than 8 weeks to promote significant changes in the microbiota, such as the establishment of beneficial taxa. In contrast, shorter treatments may result in transient and temporary changes (44, 152). The main questionnaires applied were the HAM-D and the Montgomery-Asberg Depression Rating Scale (MADRS). Some trials assessed anxiety as a secondary outcome using the STAI, Generalized Anxiety Disorder 7-item Scale, Overall Anxiety Severity and Impairment Scale (OASIS), and Brief Anxiety Scale of Tyrer (BAS). However, the results were not published or mentioned by those who evaluated it. The serum biomarkers evaluated included pro-inflammatory cytokines (IL-1β, IL-6, IL-17, and TNF-α), BDNF, and neurotransmitters. The intestinal microbiome analysis and report of adverse effects were also presented. The doses were similar to those found in the patents included in this study (10^9^ to 10^11^ CFU) and one 10^12^ CFU.

Regarding the trials completed in the database, four were published as scientific articles, and one presented only the methodological design. Only one of the trials evaluated the effects of the probiotic strain individually, *Lactobacillus plantarum* 299v at a dose of 1 × 10^10^ CFU. Another trial evaluated the effects of the association between two probiotic strains known as Probio'Stick (*Lactobacillus helveticus* R0052 and *Bifidobacterium longum* R0175) at a dose of 6 × 10^9^ CFU. The others evaluated the use of several strains in a probiotic pool, one of which was a commercial product Vivomixx^®^ (9 × 10^11^ CFU), in addition to BioKefir and a multi-strain probiotic (2 × 10^9^ CFU).

The strain administered individually did not affect HAM-D scores or inflammatory markers, but reduced serum kynurenine, resulting in a switch from the tryptophan metabolic pathway to serotonin formation. There were no serious adverse effects attributed to the probiotic, but gastrointestinal symptoms and headache were associated with the initiation of SSRI therapy (153). The Vivomixx^®^ was associated with a significant reduction in HAM-D scale scores and an increase in *Lactobacillus* bacteria, although there was no impact on BDNF levels. Administration occurred for four weeks, and the authors suggested that an eight-week supplementation could be more effective on cognitive parameters (21, 154). Treatment with the multi-strain probiotic also resulted in a significant reduction in the HAM-D score, despite some transient adverse effects such as nausea and indigestion. The combination of bacteria did not affect the levels of inflammatory markers (IL-1β, IL-6, IL-17, and TNF-α), BDNF or neurotransmitters. The antidepressant effects were attributed to the positive impact on intestinal microbiota diversity, increasing beneficial taxa (153, 155). No results were found on the effects of the association of two strains and the consumption of BioKefir.

The trials with an undefined status in the database only contained the experimental design and were conducted with *Lactobacillus* strains. The trial coordinated by Brazilian researchers used an undisclosed strain of *Lactobacillus helveticus* at a dose of 10^9^ CFU. Two trials were conducted with *Lactobacillus plantarum* PS128 (3 × 10^10^ CFU) strain in patients with depressive disorder and another in patients with depression and high levels of inflammation. There are still two trials recruiting patients to conduct studies with different aims. Both will use the Quick Inventory of Depressive Symptomatology (QIDS-RS) to assess the effects on depression. One of them aims to evaluate whether *Akkermansia muciniphila* can mitigate some of the effects related to the use of conventional therapy, such as weight gain and metabolic abnormalities. While the other will evaluate the effects of GynMDD (a multi-targeted microbiotherapy add-on) in patients who have not responded to the first line of antidepressant treatment and who are receiving a second antidepressant as an add-on. Finally, a trial not yet recruiting patients plans to administer fecal microbiota transplant capsules containing an undisclosed strain of *Lactobacillus* at 10^12^ CFU along with conventional antidepressant therapy for 8 weeks and will assess the effects using HAM-D.

In the clinical trials in which the outcomes were published, researchers pointed out methodological limitations and suggested that the different forms of presentation of depression may impact the mechanism of action of probiotics. Despite the scarcity, the trials must be conducted with rigor and high quality, as recent reviews and meta-analyses have pointed to low quality or efficacy of probiotics in clinical trials in patients with anxiety and depression, possibly due to the test design. This does not negate the benefits of probiotics. These reviews highlighted trials with few reported adverse effects and consumption for about 8 weeks, which were associated with reduced scores on validated questionnaires for anxiety and depression. Furthermore, they reinforced the importance of determining the target population and combining questionnaires with biomarker analysis to generate more reliable data (13, 156). In addition to factors inherent to the study methodology, variables inherent to the product must also be considered, as they depend on each other. The low quality of clinical studies involving probiotics can be attributed to the variability of the probiotic strains used, the different doses and formulations used, or the study design (157).

Conducting specific clinical trials with probiotics for the treatment of anxiety and depression is still considered a challenge, mainly due to the difficulties related to strain standardization and determining the appropriate dosage for each patient, considering the severity and complexity of these conditions. Clinical trials represent an advanced research stage, bringing a product's development closer to its launch on the market. Considering the factors that can define a quality clinical trial, developing probiotics to treat anxiety and depression still depends on conducting robust clinical trials with a well-defined experimental design and adequate selection and characterization of the strains. These trials should be conducted on patients who present the aforementioned conditions, ensuring the product's efficacy, safety, and stability.

Some inherent limitations of this study should also be considered when interpreting the results. The scope of the study was restricted to patents filed in a specific database (Espacenet, the EPO patent database), which may have excluded records from underrepresented countries. An additional limitation arises from the methodological heterogeneity among the cited preclinical studies, which exhibited significant variations in animal models, dosages, and endpoints, thus complicating conclusive extrapolations to humans. Furthermore, most of the identified patents focused on well-characterized bacterial strains (e.g., *Lactobacillus* and *Bifidobacterium*), potentially overlooking the therapeutic potential of less explored microorganisms. Finally, the lack of *in vivo* analyses in diverse human populations (stratified by age, sex, and comorbidities) limits the generalizability of the findings, highlighting the need for translational studies to validate functional efficacy and clinical applicability.

## 5 Conclusion

Patent trends in microbiology and neuroscience present several significant findings. The analysis of patents with clinical trial descriptions and discussions with relevant scientific publications highlights important advances in the field, but also reveals gaps and challenges that limit the translation of these results into clinical practice. The gradual increase in patent publications related to the use of probiotics in psychiatric disorders over the years, with a notable increase since 2019, reflects the increased interest in novel therapeutic strategies, such as modulation of the gut-brain-microbiota axis. China has emerged as the main contributor to patent publications, followed by South Korea, the World Intellectual Property Organization (WIPO), and the European Patent Organization (EPO). Despite this, China was not among the countries that advanced to clinical trials. This demonstrates that the volume of clinical trials conducted in the country is still below the global average standard. Certainly, China and other depositing countries have invested in economic incentives, development, and innovation policies to overcome these limitations in the short and medium term.

The results observed in the patents suggest that probiotics exert therapeutic effects by modulating neurotransmitters, reducing neurogenic inflammation, and regulating the hypothalamic-pituitary-adrenal (HPA) axis. These mechanisms appear to be crucial for the treatment of conditions such as anxiety and depression. However, they require more robust validation, such as in clinical trials. Although some clinical trials are being conducted, many methodological limitations are observed, such as small sample size, lack of dose standardization, and absence of biomarkers that allow a consistent correlation of behavioral changes with biological mechanisms. Addressing these gaps could favor the implementation of more effective protocols in clinical practice. This could result in lower costs in subsequent studies involving other strains and, collectively, lead to the standardization of a treatment guideline.

Advances in omics and bioinformatics are emerging as promising areas in this field of study as they offer the possibility of identifying microbiological profiles associated with better therapeutic responses, allowing the development of targeted interventions. This indicates that, with the growth of studies on the therapeutic application of probiotics and biotechnological development, it may be possible to target therapies, considering variables such as the basal composition of the microbiota, individual genetics, and specific characteristics of each psychiatric disorder. Furthermore, exploring innovative vehicles such as functional foods and highly bioavailable pharmaceutical formulations may improve patient adherence and strain stability during administration and attract clinical, academic, and commercial interests. Technological investment in these probiotic formulations is the evolving trend.

Strains of *Bifidobacterium* and *Lactobacillus* were identified as the most frequently patented. Species from these genera are more widely studied and recognized for their potential health benefits, especially in regulating the intestinal microbiota, neuroendocrine, and redox balance, and reducing neuroinflammation, indicating a significant focus on therapeutic applications. These technologies point to effects that may represent a significant advance in the psychiatric clinical context. The anxiolytic and antidepressant potential of strains from other genera is expected in future research. We are far from the definitive endpoint in this field of investigation. The present review insistently reinforces this fact.

In this study, as prospects indicate the likelihood that probiotic therapy will advance in two main directions. First, more robust translational studies will be essential for establishing standardized doses and protocols to connect experimental findings with clinical applications. Second, the development of combination products, which integrate probiotics with other therapeutic interventions, such as pre- and post-biotics or even with classic drug treatments, may offer an integrated approach to complex psychiatric conditions. Therefore, the use of probiotics for the treatment of anxiety and depression is on the rise, driven by the increasing understanding of the underlying mechanisms, by academic and industrial interest, by the current appeal for alternative treatments, and by the exponential increase in the number of cases of diagnoses of these diseases. However, the success of these technologies will depend on overcoming methodological, regulatory, and commercial barriers, requiring a collaborative effort between researchers, industries, and policymakers to register products with therapeutic applications. The integrated patent analysis highlights a convergence between probiotic therapy and neuroscience, suggesting a growing trend in using microorganisms as therapeutic interventions. Overall, these results provide insights into the evolving landscape of patent activity in probiotic therapy for anxiety and depression, indicating a dynamic interplay between scientific advances, technological innovation, and mental health applications.

## Data Availability

The original contributions presented in the study are included in the article/Supplementary material, further inquiries can be directed to the corresponding author.
