# Supplementary material for: Probiotics as technological innovations in psychiatric disorders: patents and research reviews
Source: Front Nutr. 2025 Apr 24;12:1567097. doi: 10.3389/fnut.2025.1567097 (PMC12060171; doi:10.3389/fnut.2025.1567097)
Supplement: Supplementary file 1 [file Data_Sheet_1.pdf]

Probiotics as Technological Innovations in Psychiatric Disorders: Patents and Research Reviews

Supplementary material – Table 1

Publication number	Year	Country	Main probiotic strain	ConcentrationCFU/mL)	Effect	Model and assays that were tested	Preparations
WO2004098622A2<br>WO2004098622A3	2003	WO	*Bifidobacterium infantis 35624*	-	Antidepressant effect:<br>↓HAMD and BDI scores. HAMD score from 23 to 4;<br>↓blood pro-inflammatory cytokines (SIL-6R and IL-8) levels.	Case report (depressed patients); assays:<br>1. HAM-D.	Milk suspension
EP3072398A1<br>EP3072398B1	2008	EP	*Bifidobacterium longum ATCC BAA-999*	10^10 CFU/mL	Anxiolytic effect:<br>↑entries in light compartment;<br>↑time spent in the light compartment;<br>↓latency to step down from the pedestal;<br>↑BDNF levels.	In vivo (BALB/c mice); assays:<br>1. light-dark box;<br>2. step-down test.	Food and fermented food
US11225641B2<br>US2020325548A1	2009	US	*Bifidobacterium AH171*	-	Anxiolytic and antidepressant effects:<br>↓freezing;<br>↓number of buried marbles;<br>↑latency to immobility;<br>↓immobility time.	In vivo (mice); assays:<br>1. fear conditioning test (anxiety);<br>2. marble-burying test (anxiety);<br>3. tail suspension (depression).	Pharmaceutical composition and food
US2018271919A1	2015	US	*Bifidobacterium bifidum W23*	5×10^9 CFU/day	Antidepressant effect:<br>↓immobility time;<br>↓cognitive reactivity in response to bad mood.	In vivo (rat); retention stress model; assays:<br>1. swimming forced.<br><br>Clinical trial (healthy patients):<br>1. LEIDS-R.	Milk suspension<br><br>Pharmaceutical composition and food

KR20200101950A	2017	KR	*Lactobacillus plantarum*	10^9 CFU/day	Anxiolytic and antidepressant effects: ↑entries in open arms (LP12407 and LP12418); ↓immobility time.	In vivo (Swiss mice); chronic stress-induced model; assays: 1. elevated plus-maze (anxiety); 2. swimming forced (depression).	0.9% NaCl vehicle<br>Pharmaceutical composition and food
CN108715822A CN108715822B	2018	CN	*Lactobacillus rhamnosus TF318*	2×10^10 CFU/mL	Antidepressant effect: ↓drooping of the eyelids; ↑circle exit rate; ↑serotonin levels; ↓corticosterone levels; ↓blood pro-inflammatory cytokines (TNF-α and IL-1β) levels; ↓blood oxidative stress markers (NO); ↓lipid peroxidation (MDA).	In vivo (ICR mice); reserpine-induced depression model; assays: 1. degree of eyelid drooping; 2. circle rate outside = (the total number of mice - remained inside The number of mice on the white paper).	Bacterial suspension. Food.
CN108949640A CN108949640B	2018	CN	*Bifidobacterium breve CCFM1025*	5×10^9 CFU/mL	Antidepressant effect: ↑preference for sugar water; ↓immobility time; ↓time in plartform with electric shock; ↑serotonin levels; ↑BNDF levels; ↓blood corticosterone levels.	In vivo (C57BL/6J mice); assays: 1. sugar water preference; 2. tail suspension; 3. swimming forced; 4. open field test; 5. platform test.	Bacterial suspension and fermented food
WO2020037533A1	2018	WO	*Bifidobacterium longum subsp. infantis CCFM687*	5×10^9 CFU/mL	Antidepressant effect: ↑preference for sugar water; ↓immobility time; ↓time in plartform with electric shock; ↑serotonin levels; ↑BNDF levels.	In vivo (C57BL/6J mice); chronic unpredictable stress depression model; assays: 1. sugar water preference; 2. tail suspension; 3. swimming forced; 4. platform test.	5% sterilized skim emulsion Fermented food

This is a provisional file, not the final typeset article

KR20210097716A	2018	KR	*Christensenella minuta DSM 32891, and Christensenellaceae bacteria*	10^9 CFU/mL	Anxiolytic and antidepressant effects: ↓immobility time; ↑sucrose preference; ↑serotonin levels.	In vivo (C57BL/6J mice); acute social stress model; assays: 1. sucrose preference (anxiety); 2. tail suspension (depression).	Food Skim milk 10%
KR101862051B1	2018	KR	*Bifidobacterium adolescentis IM38*	5x10^9 CFU 10^9 CFU	Anxiolytic effect: ↑entries in open arms; ↑time in open arms; ↓blood pro-inflammatory cytokines (TNF-α, IL-6) levels.	In vivo (C57BL/6 mice); containment-induced anxiety model; assays: 1. elevated plus-maze.	Bacterial suspension<br><br>Pharmaceutical composition and food compositions
KR20210102916A	2018	KR	*Lactobacillus plantarum LP12151*	10^9 CFU	Antidepressant effect: ↓immobility time.	In vivo (Swiss mice); chronic stress model; assays: 1. swimming forced.	Pharmaceutical composition and food.
KR20220119055A	2019	KR	*Clostridium NCIMB 43454*	10^9 CFU/mL	Anxiolytic and antidepressant effects: ↑entries in open arms; ↑time in open arms; ↓immobility time; ↑serotonin levels; ↑tryptophan levels; ↑BDNF levels.	In vivo (BALB/c mice); assays: 1. elevated plus-maze (anxiety); 2. swimming forced (depression).	Food
WO2020238870A1	2019	WO	*Pediococcus acidilactici CCFM6432*	5×10^9 CFU/mL	Anxiolytic and antidepressant effects: ↑entries in open arms; ↑time in open arms; ↑latency to immobility; ↓immobility time; ↓HPA-axis activity; ↓blood corticosterone levels; ↓blood pro-inflammatory cytokines (TNF-α, IL-1β and IL-6) levels.	In vivo (C57BL/6J mice); unpredictable chronic stress model; assays: 1. elevated plus-maze (anxiety); 2. tail suspension (depression).	Bacterial suspension. Functional foods, medicines and health products

CN110066753A CN110066753B	2019	CN	*Lactobacillus plantarum DP189*	2×10^10 CFU/mL	Antidepressant effect: ↓latency to find the platform; ↑crossings; ↑BDNF levels; ↓ACTH levels.	In vivo (Wistar rats); administration of corticosterone; assays: 1. water maze test.	Food and pharmaceutical composition
JP2021045054A	2019	JP	*Lactobacillus herbeticas NITE BP-01671*	10^10 CFU/g	Antidepressant effect: ↓STAI score.	Clinical trial (depressed patients): 1. STAI.	Skim milk 10% Fermented food
WO2021008149A1	2019	WO	*Akkermansia Muciniphila*	-	Antidepressant effect: ↑total movement distance; ↓immobility time.	In vivo (C57BL/6 mice); assays: 1. open field test; 2. tail suspension; 3. swimming forced.	Sterile PBS vehicle Bacterial suspension
CN110279119A	2019	CN	*Streptococcus thermophilus S709, Lactobacillus paracasei L578, Lactobacillus helveticus L551, and Streptococcus thermophilus S709*	10^9 CFU/g	Antidepressant effect ↓HAMA score.	Clinical trial (depressed patients): 1. Hamilton Anxiety Index (HAMA).	Solution Food
CN116744806A	2020	CN	*Bifidobacterium animalis subsp. lactis HEM20-01*	5×10^7 CFU/ml	Antidepressant effect: ↑preference for sugar water; ↓latency to feeding; ↓ latency to lick; ↑lick time; ↑latency to immobility; ↓immobility time; ↓pro-inflammatory cytokines (IL-1β, IL-6 , TNF-α and IFN-γ) in hippocampus; ↑blood serotonin levels; ↓blood corticosterone levels.	In vivo (BALB/c mice); assays: 1. sugar water preference and; 2. change in feed supply; 3. spray test (self-management behavior); 4. tail suspension; 5. forced swimming.	Pharmaceutical composition and food

This is a provisional file, not the final typeset article

CN111743159A<br>CN111743159B	2020	CN	*Bifidobacterium breve CCFM1025, Bifidobacterium longum subsp. infantis CCFM687, and Pediococcus lactis CCFM6432*	10^9 CFU	Antidepressant and anxiolytic effects:<br>↑sucrose preference;<br>↓immobility time;<br>↑entries in open arms;<br>↑time in open arms;<br>↑serotonin levels;<br>↓blood ACTH levels;<br>↓blood corticosterone levels.	In vivo (C57BL/6J mice); unpredictable chronic stress model; assays:<br>1. sucrose preference (depression);<br>2. swimming forced (depression);<br>3. elevated plus-maze (anxiety).	Skim milk 10%
CN111560331A<br>CN111560331B	2020	CN	*Lactobacillus paracasei nbk-LC16*	10^8 CFU/mL	Antidepressant effect:<br>↑distance covered;<br>↑preference for sugar water;<br>↑serotonin levels;<br>↓blood CRH levels;<br>↓corticosterone levels.	In vivo (SD rats); CUMS model; assays:<br>1. open field test;<br>2. sugar water preference.	Lyophilized powder and bacterial suspension
CN111728111A<br>CN111728111B	2020	CN	*K-KLJ (Lactobacillus and Bifibacterium species)*	In solid drink containing 1.8-8.5×10^8 CFU/g.<br>In yogurt, 2.7×10^8 CFU/g.<br>In drink, 2.8×108 CFU/g.<br>In Yogurt block, 5.5×10^9 CFU/g.	Anxiolytic and antidepressant effects:<br>↑time spent in the light compartment;<br>↓immobility time.	In vivo (Kunming mice); assays:<br>1. light-dark box;<br>2. tail suspension.	Food
CN112914107A<br>CN112914107B	2020	CN	*Probiotic strain Ls17*	10^9 CFU/mL	Antidepressant effect:<br>↑sucrose preference;<br>↓immobility time.	In vivo (BALB/c mice); They were germ-free initially; Afterwards, he received a fecal microbiota transplant from depressed patients; assays:<br>1. sucrose preference;<br>2. tail suspension.	Food, medicine or health care products

CN113512514A<br>CN113512514B	2021	CN	*Lactococcus lactis*	1×10^9 CFU/day	Antidepressant and anxiolityc effects:<br>↓immobility time;<br>↑distance covered;<br>↑serotonin levels;<br>↑BDNF levels;<br>↓blood corticosterone levels.	In vivo (BALB/c mice); CUMS model; assays:<br>1. tail suspension (depression);<br>2. forced swimming (depression);<br>3. open field test (anxiety).	Lyophilized powder and pharmaceutical composition
CN113832086A<br>CN113832086B	2021	CN	*Bifidobacterium bifidum BXM0*	10^9CFU/mL	Antidepressant effect:<br>↓immobility time;<br>↑GABA production.	In vivo (Kunming mice); assays:<br>1. swimming forced.	Bacterial suspension
CN112999246A	2021	CN	*Bifidobacterium longum, Lactobacillus helveticus, Lactobacillus rhamnosus, and Lactobacillus acidophilus*	5×10^9 CFU/mL	Antidepressant effect:<br>↓immobility time;<br>↑blood serotonin levels;<br>↓HAMD and BDI scores.	In vivo (C57BL/6 mice); assays:<br>1. tail suspension;<br>2. forced swimming.<br><br>Clinical trial (depressed patients):<br>1. HAM-D;<br>2. BDI.	Food, health products or medicine
CN113322202A<br>CN113322202B	2021	CN	*Akkermansia muciniphila AKK-JWA*	1×10^9 CFU/ml	Anxiolytic and antidepressant effects:<br>↑total movement distance;<br>↑time in central area;<br>↓immobility time.	In vivo (C57BL/6 mice); assays:<br>1. open field test (anxiety);<br>2. tail suspension (depression).	Bacterial suspension
CN114081184A<br>CN114081184B	2021	CN	*Lactobacillus plantarum Lp3a, Lactobacillus paracasei LPC45, and Bifidobacterium breve BB033*	2x10^11 CFU/g	Antidepressant effect:<br>↑latency to immobility;<br>↓immobility time.	In vivo (ICR mice); assays:<br>1. tail suspension;<br>2. forced swimming.	Food, health products or medicines
WO2023079036A1	2021	WO	*Lactobacillus salivarius, Lactobacillus camelliae, and*	10^10 CFU	Antidepressant effect:<br>↓immobility time.	In vivo (C57B1/6 mice); assays:<br>1. swimming forced.	Pharmaceutical composition and food

			Bifidobacterium ruminantium				
WO2023156945A1	2022	WO	*Lacticaseibacillus rhamnosus HN001 and/or Bifidobacterium animalis subsp. lactis HN019*	6x10^9 CFU/day	Anxiolytic and antidepressant effects:<br>↑distance covered;<br>↑serotonin levels;<br>↓blood corticosterone levels;<br>↓blood pro-inflammatory cytokines (TNF-α, IL-1β, IL-18 and IL-6) levels.	In vivo (Rats); CUMS model; assays:<br>1. open field test (depression);<br>2. elevated plus-maze (anxiety).	Food
CN115838653A	2022	CN	*Lactobacillus plantarum GM11*	2×10^9 CFU/mL	Anxiolytic and antidepressant effects:<br>↑time in open arms;<br>↑time in central area;<br>↑preference for sugar water;<br>↓immobility time;<br>↑blood serotonin levels;<br>↑blood BDNF levels;<br>↓blood corticosterone levels;<br>↓pro-inflammatory cytokines (TNF-α and IL-1β) levels.	In vivo (SD rats); CUMS model; assays:<br>1. elevated plus-maze (anxiety);<br>2. open field test (anxiety);<br>3. sugar water preference (anxiety);<br>4. swimming forced (depression).	Food
CN115948281A	2022	CN	*Lactobacillus plantarum*	10^8 CFU/mL	Antidepressant effect:<br>↓immobility time;<br>↑sucrose preference;<br>↑serotonin levels;<br>↑BDNF levels;<br>↓corticosterone levels;<br>↓blood pro-inflammatory cytokines (IL-1β and IL-6) levels;<br>↑anti-inflammatory cytokines (IL-10) levels;<br>↑antioxidant markers (GSP-PX, SOD, T-AOC and Nrf-2);<br>↓lipid peroxidation (MDA).	In vivo (C57BL/6J mice); CUMS model; assays<br>1. tail suspension test;<br>2. swimming forced;<br>3. sucrose preference.	Food

CN116064326A	2022	CN	*Bifidobacterium animalis subsp. lactis GBW8051*	10^8 CFU/mL	Antidepressant effect:<br>↑preference for sugar water;<br>↓immobility time;<br>↑serotonin levels;<br>↑BNDF levels;<br>↓blood corticosterone levels.	In vivo (C57BL/6J mice); assays:<br>1. sugar water preference;<br>2. tail suspension;<br>3. swimming forced.	Bacterial suspension
CN114540245A<br>CN114540245B	2022	CN	*Lactobacillus rhamnosus CCFM1228*	1×10^9 CFU/mL<br>5×10^9 CFU/mL	Antidepressant effect:<br>↑time in central area;<br>↓immobility time;<br>↓number of buried marbles;<br>↑serotonin levels;<br>↓blood CRH levels;<br>↑BDNF levels.	In vivo (C57BL/6J mice); CUMS model; assays:<br>1. open field test;<br>2. tail suspension;<br>3. swimming forced;<br>4. marble-burying test.	Bacterial suspension.<br>Food
CN114410547A<br>CN114410547B	2022	CN	*Lactobacillus pentosus LPQ1*	1 ×10^9 CFU/mL	Antidepressant effect:<br>↑preference for sugar water;<br>↓swimming time;<br>↓immobility time;<br>↑distance covered;<br>↓blood pro-inflammatory cytokines (TNF-α and IL-6) levels;<br>↑blood serotonin levels.	In vivo (BALB/c mice); CUMS model; assays:<br>1. sugar water preference;<br>2. swimming forced;<br>3. tail suspension;<br>4. open field test.	Lyophilized powder<br>Food and pharmaceutical composition
CN114774318A<br>CN114774318B	2022	CN	*Lactobacillus paracasei LC86*	5x10^8 CFU/g	Anxiolytic and antidepressant effects:<br>↑entries in open arms;<br>↑time in open arms;<br>↓immobility time;<br>↑serotonin levels.	In vivo (120KM mice); assays:<br>1. elevated plus-maze (anxiety);<br>2. tail suspension (depression).	Lyophilized powder
CN116121131A	2022	CN	*Bifidobacterium longum subsp. longum LF03*	10^5 CFU/mL<br>10^6 CFU/mL	Antidepressant effect:<br>↑distance covered;<br>↓immobility time;<br>↓monoamine oxidase levels.	In vivo (zebrafish); reserpine-induced depression model; assays:<br>1. degree of activity: distance covered, manic time, active time and rest time.	Bacterial suspension

This is a provisional file, not the final typeset article

CN114947135A<br>CN114947135B	2022	CN	*Lactobacillus rhamnosus and Lactobacillus fermentum*	10^8 CFU/mL	Anxiolytic effect:<br>↑time in open arms;<br>↑time spent in the light compartment.	In vivo (ICR mice); assays:<br>1. elevated plus-maze;<br>2. light-dark box.	Pharmaceutical composition and food
CN116474002A	2023	CN	*Akkermansia muciniphila AM06*	10^10 CFU/mL	Antidepressant and anxiolytic effects:<br>↑time in central area;<br>↑time in open arms;<br>↑entries in open arms;<br>↑blood serotonin levels;<br>↓blood CRH levels;<br>↓pro-inflammatory cytokines (IL-8) levels.	In vivo (C57BL/6 mice); assays:<br>1. open field test (depression);<br>2. elevated plus-maze (anxiety).	Bacterial suspension
CN117143760A	2023	CN	*Lactobacillus rhamnosus*	-	Antidepressant effect:<br>↑time in open arms;<br>↑time in central area;<br>↑latency to immobility;<br>↓immobility time;<br>↓HPA-axis activity;<br>↓blood corticosterone levels;<br>↓blood pro-inflammatory cytokines (TNF-α and IL-1β) levels;<br>↑blood anti-inflammatory cytokines (IL-10) levels;<br>↑gene expression of characteristic indicators of glial cell M2 polarization;<br>↑tryptophan hydroxylase 1 (TPH) genes.	In vivo (mice); CUMS model; assays:<br>1. open field test;<br>2. elevated plus-maze;<br>3. swimming forced;<br>4. tail suspension.	Lyophilized powder
CN116751726A	2023	CN	*Weizwinia coagulans MAT411*	5×10^9 CFU/mL	Antidepressant effect:<br>↑preference for sugar water;<br>↑serotonin levels;<br>↑BDNF levels.	In vivo (SD rat); chronic corticosterone-induced model of depression; assays:<br>1. sugar water preference.	Pharmaceutical composition

CN117338822A	2023	CN	*Bacillus subtilis BS02*	10^6 CFU/mL	Antidepressant effect: ↑distance covered; ↑average speed of movement; ↓immobility time; ↑serotonin levels.	In vivo (zebrafish); reserpine-induced depression model; assays: 1. degree of activity: distance of movement, average speed of movement and time of immobility.	Bacterial suspension
CN117547558A	2023	CN	*Bifidobacterium adolescentis BAS05*	1×10^6 CFU/mL	Antidepressant effect: ↑distance covered; ↑average speed of movement; ↓immobility time; ↑serotonin levels.	In vivo (zebrafish); reserpine-induced depression model; assays: 1. degree of activity: distance of movement, average speed of movement and time of immobility.	Bacterial suspension
CN117205237A	2023	CN	*Bifidobacterium animalis subsp. lactis BL03*	1×10^6 CFU/mL	Antidepressant effect: ↑distance covered; ↑average speed of movement; ↓immobility time; ↑serotonin levels.	In vivo (zebrafish); reserpine-induced depression model; assays: 1. degree of activity: distance of movement, average speed of movement and time of immobility.	Bacterial suspension
CN117398416A	2023	CN	*Bifidobacterium longum LF04*	1×10^6 CFU/mL	Antidepressant effect: ↑distance covered; ↑average speed of movement; ↓immobility time; ↑serotonin levels.	In vivo (zebrafish); reserpine-induced depression model; assays: 1. degree of activity: distance of movement, average speed of movement and time of immobility.	Bacterial suspension
CN117205236A	2023	CN	*Clostridium butyricum LS05*	1×10^6 CFU/mL	Antidepressant effect: ↑distance covered; ↑average speed of movement;	In vivo (zebrafish); reserpine-induced depression model; assays:	Bacterial suspension

This is a provisional file, not the final typeset article

					↓immobility time;<br>↑serotonin levels.	1. degree of activity: distance of movement, average speed of movement and time of immobility.	
CN117379464A	2023	CN	*Lactobacillus helveticus LH05*	1×10^6 CFU/mL	Antidepressant effect:<br>↑distance covered;<br>↑average speed of movement;<br>↓immobility time;<br>↑serotonin levels.	In vivo (zebrafish); reserpine-induced depression model; assays:<br>1. degree of activity: distance of movement, average speed of movement and time of immobility.	Bacterial suspension
CN117398417A	2023	CN	*Lactobacillus paracasei E6*	10^6 CFU	Antidepressant effect:<br>↑distance covered;<br>↑average speed of movement;<br>↓immobility time;<br>↑serotonin levels.	In vivo (zebrafish); reserpine-induced depression model; assays:<br>1. degree of activity: distance of movement, average speed of movement and time of immobility.	Bacterial suspension
CN117205238A	2023	CN	*Lactobacillus rhamnosus NX-2*	10^6 CFU/mL	Antidepressant effect:<br>↑distance covered;<br>↑average speed of movement;<br>↓immobility time;<br>↑serotonin levels.	In vivo (zebrafish); reserpine-induced depression model; assays:<br>1. degree of activity: distance of movement, average speed of movement and time of immobility.	Bacterial suspension
CN117398418A	2023	CN	*Lactobacillus salivarius LF01*	10^6 CFU/mL	Antidepressant effect:<br>↑distance covered;<br>↑average speed of movement;<br>↓immobility time;<br>↑serotonin levels.	In vivo (zebrafish); reserpine-induced depression model; assays:<br>1. degree of activity: distance of movement, average speed of	Solution

						movement and time of immobility.	
CN116555129A CN116555129B	2023	CN	*Lactobacillus gasseri BDUP*	10^9 CFU/mL	Antidepressant effects: ↓immobility time; ↑GABA levels.	In vivo (Kinming mice); CUMS model; assays: 1. tail suspension; 2. forced swimming.	Food
CN116676226A	2023	CN	*Lactobacillus plantarum LP-28*	250 ml of the yogurt = 10^8 CFU/mL.	Anxiolytic effect: ↓HAMA score; ↑GABA levels.	Clinical trial (anxious patients): 1. HAMA.	Fermented milk
CN116327813A	2023	CN	*Lactobacillus animalis JCM 5670*	10^9 CFU	Antidepressant effect: ↑preference for sugar water; ↓immobility time.	In vivo (SD rats); assays: 1. sugar water preference; 2. tail suspension; 3. swimming forced.	Bacterial suspension
CN117530964A	2023	CN	*Pasteurella enterica*	1×10^9 CFU/ml	Antidepressant effect: ↑entries in central area; ↑time in central area; ↓immobility time; ↑social score.	In vivo (C57BL/6 mice); assays: 1. open field test; 2. tail suspension; 3. forced swimming forced; 4. social interaction test.	Bacterial suspension
CN117297098A	2023	CN	*Bacillus Coagulans, Bifidobacterium breve, Bifidobacterium Infantis, Bifidobacterium Longum, Lactobacillus Casei, Lactobacillus Helveticus, Lactobacillus Paracasei, Lactobacillus Plantarum, and*	10^10 CFU	Antidepressant effect: ↓depression detection score.	Clinical trial (depressed patients): 1. 62-question questionnaire.	Food

This is a provisional file, not the final typeset article

			Lactobacillus Rhamnosus				

Notes: Hamilton Depression Rating Scale (HAM-D), Leiden Index of Depression Sensitivity-Revised (LEIDS-R), Chronic unpredictable mild stress (CUMS), Beck Depression Inventory (BDI), Hamilton Anxiety Index (HAMA), State-Trait Anxiety Initiative (STAI), γ-aminobutyric acid (GABA), tumor necrosis factor alpha (TNF-α), interleukines (IL), glutathione peroxidase (GPX), superoxide dismutase (SOD), total antioxidant capacity (T-AOC), nuclear factor erythroid 2–related factor 2 (Nrf-2), malonaldehyde (MDA), nitric oxide (NO), interferon-γ (IFN-γ), brain-derived neurotrophic factor (BDNF), adrenocorticotropic hormone (ACTH), corticotropin releasing hormone (CRH).
